# Single-molecule live-cell imaging visualizes parallel pathways of prokaryotic nucleotide excision repair

**DOI:** 10.1038/s41467-020-15179-y

**Published:** 2020-03-20

**Authors:** Harshad Ghodke, Han Ngoc Ho, Antoine M. van Oijen

**Affiliations:** 1 Molecular Horizons and School of Chemistry and Molecular Bioscience, University of Wollongong, Wollongong, NSW 2522 Australia; 2 Illawarra Health and Medical Research Institute, Wollongong, NSW 2522 Australia

**Keywords:** DNA repair enzymes, Escherichia coli, Fluorescence imaging, Single-molecule biophysics, Bacteriology

## Abstract

In the model organism *Escherichia coli*, helix distorting lesions are recognized by the UvrAB damage surveillance complex in the global genomic nucleotide excision repair pathway (GGR). Alternately, during transcription-coupled repair (TCR), UvrA is recruited to Mfd at sites of RNA polymerases stalled by lesions. Ultimately, damage recognition is mediated by UvrA, followed by verification by UvrB. Here we characterize the differences in the kinetics of interactions of UvrA with Mfd and UvrB by following functional, fluorescently tagged UvrA molecules in live TCR-deficient or wild-type cells. The lifetimes of UvrA in Mfd-dependent or Mfd-independent interactions in the absence of exogenous DNA damage are comparable in live cells, and are governed by UvrB. Upon UV irradiation, the lifetimes of UvrA strongly depended on, and matched those of Mfd. Overall, we illustrate a non-perturbative, imaging-based approach to quantify the kinetic signatures of damage recognition enzymes participating in multiple pathways in cells.

## Abstract (web summary)

In Escherichia coli, the UvrAB damage sensor recognizes helix-distorting lesions by itself or via Mfd bound to stalled RNA polymerase. Here authors use single-molecule fluorescence imaging to quantify the kinetic signatures of interactions of UvrA with Mfd and UvrB in live cells.

## Introduction

**Fig. 1:**
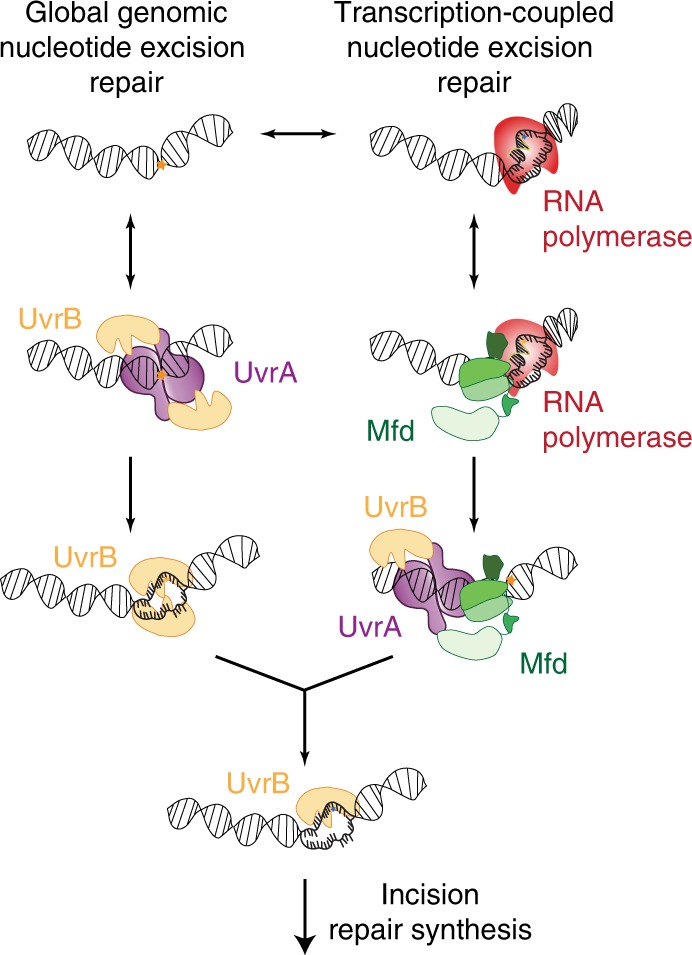
Nucleotide excision repair in *Escherichia coli*. Damage detection in nucleotide excision repair in *E. coli* proceeds via global damage surveillance executed by UvrA_2_(B), and RNA polymerase (RNAP) transcribing damaged template DNA. The UvrA dimer loads UvrB, which verifies the presence of DNA damage in a strand-specific manner. Alternately, stalled elongation complexes at the site of DNA damage are rescued by the transcription-repair coupling factor Mfd, which in turn recruits UvrA_2_(B) to the site of the stalled RNAP. This is followed by strand-specific loading of UvrB at the site of the lesion. Following damage verification by UvrB, a single-stranded patch of DNA containing the damage is incised by the UvrC endonuclease. This is followed by repair synthesis and ligation coordinated by UvrD, PolI and LigA.

Across the various domains of life, the recognition and repair of bulky, helix-distorting lesions in chromosomal DNA is coordinated by nucleotide excision repair (NER) factors. Damage detection occurs in two stages during GGR: a dedicated set of damage surveillance factors^1–3^ (namely the prokaryotic UvrA, and the eukaryotic ultraviolet DNA damage binding protein UV-DDB, XPC, XPA and homologs) constantly survey genomic DNA for lesions. At sites of putative DNA damage, these damage recognition factors load specific DNA damage verification factors (UvrB in prokaryotes, TFIIH and homologs in eukaryotes) that unwind the DNA and verify the location of the damage with nucleotide resolution (Fig. 1a) (reviewed in refs. ^2,3^). Subsequently, specialized endonucleases (the prokaryotic UvrC and homologs, and the eukaryotic XPF-ERCC1/XPG and homologs) are recruited to the site of the DNA, resulting in cleavage of a single-stranded DNA (ssDNA) patch containing the lesion (reviewed in refs. ^2,3^).

In several studied organisms (barring certain archaea^4,5^), strand-specific removal of DNA damage also occurs following stalling of RNA polymerase (RNAP) at sites of lesions (reviewed in ref. ^6^). In this case, a ternary elongation complex (TEC) of RNAP that is unable to catalyse nascent RNA synthesis manifests as an ultra-stable protein-DNA roadblock. Dedicated transcription-repair coupling factors including the prokaryotic Mfd (and eukaryotic homologs Rad26/CSB) remodel these TECs and terminate transcription^7–11^. In prokaryotes, Mfd subsequently recruits the UvrA_2_B complex to the stall site (Fig. 1)^9,11,12^. Similarly, in eukaryotes, CSB recruits the TFIIH complex^13^ to the site of stalled RNAPII. DNA repair triggered at stalled TECs is termed transcription-coupled repair (TCR). Consequently, the rate of removal of UV-induced lesions from the transcribed strand during TCR is enhanced compared to that from non-transcribed DNA in GGR^14–19^. This observation has sparked several studies targeted at understanding the mechanistic basis of rate enhancement^12,20,21^.

A diverse set of intermediates is readily formed in vitro—ranging from a translocating RNAP-Mfd complex, arrested RNAP-Mfd-UvrA_2_ and the complete Mfd-UvrA_2_-UvrB handoff complex in the presence of both UvrA and UvrB^12,20,22^. To determine the physiological relevance of these intermediates, we visualized Mfd in cells and quantified its lifetime in the TCR reaction^23^. A recent study characterizing the behaviour of fluorescently tagged UvrA in live cells failed to detect its participation in TCR^24^. Therefore, in vitro studies notwithstanding, how TCR is orchestrated by UvrA in cells remains unclear.

In this work, we revisited this question and applied high-resolution single-molecule imaging methods that permit accurate measurements of DNA-binding lifetimes over a broad timescale ranging from a few hundred milliseconds to several minutes^25^. We asked the question: what is the lifetime of interactions of UvrA with Mfd and UvrB on DNA? To answer this question, we visualized fluorescently tagged UvrA and measured the lifetimes of interactions with DNA in wild-type and TCR-deficient cells. We find that UvrA is long lived on DNA in the absence of UvrB and Mfd, and that its dissociation is promoted by UvrB in cells. The cellular concentration of UvrA relative to UvrB strongly influences its binding lifetime in interactions with DNA-bound Mfd. The binding lifetimes of Mfd-UvrA interactions are only detected under conditions of limiting UvrB in the absence of exogenous DNA damage, suggesting a role for UvrB in resolving Mfd-UvrA intermediates. Exposure to UV light leads to an increase in the binding lifetime of UvrA in TCR-deficient cells. In contrast, in TCR-proficient cells, the DNA-bound lifetimes of UvrA and Mfd are identical, and drop upon UV exposure suggesting the formation of an Mfd-UvrA intermediate in TCR. Together, these studies characterize the interaction of UvrA with Mfd in live cells. Here, we establish a comprehensive framework for characterizing the binding kinetics of DNA repair proteins participating in multiple parallel pathways in vivo using non-perturbative, single-molecule imaging approaches.

## Results

### Imaging of UvrA-YPet in the absence of exogenous DNA damage

The NER reaction occurs in five discrete stages: damage recognition, damage verification, incision, repair synthesis and ligation (reviewed in ref. ^2^). UvrA mediates damage recognition by itself and by interacting with Mfd^1^ by surveying the genome constantly for the presence of DNA damage or Mfd-bound TECs. Following detection of a putative damage site, damage verification is then orchestrated by the damage verification enzyme UvrB^26^ (Fig 1).

**Fig. 2:**
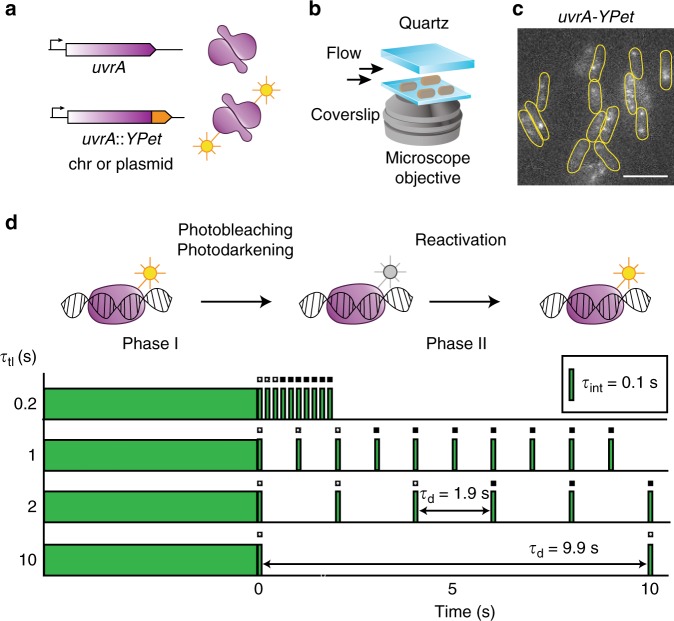
Construction of *uvrA-YPet* and imaging of UvrA-YPet. **a** A chromosomal fusion of UvrA to the yellow fluorescent protein (YPet) under the native *uvrA* locus was created using λ Red recombination in MG1655 cells. In a second approach, the *uvrA-YP*et allele was expressed under the native *uvrA* promoter from a low-copy plasmid in Δ*uvrA* cells. **b** Cells expressing fluorescent UvrA-YPet were grown to early exponential phase and loaded in a flow cell. Cells were imaged under constant supply of aerated growth medium. **c** Fluorescence images of UvrA-YPet reveal a mixture of foci and diffuse cellular background signal. Scale bar represents 5 μm. Cell outlines are provided as a guide to the eye. **d** Schematic of interval imaging approach employed to measure the off rates of fluorescently tagged proteins in cells. Each acquisition is collected in two phases. In the first phase, fluorescent signal is bleached to enable observation of single fluorescent YPet molecules. In the second phase, a dark frame *τ*_d_ is introduced such that the time-lapse time *τ*_tl_ = *τ*_d_ + *τ*_int_, where *τ*_int_ is the integration time (100 ms). In this phase, the lifetimes of individual binding events of UvrA-YPet molecules are measured and combined to obtain a cumulative residence time distribution.

We visualized fluorescently tagged UvrA in live cells to characterize the kinetic basis of damage surveillance and UvrB loading. We replaced the *uvrA* gene with a C-terminal fusion of *uvrA* to the gene for the yellow fluorescent protein (YPet^27^) in MG1655 cells using λ Red recombination (Fig. 2a)^28^. This strategy enabled observation of UvrA-YPet expressed from the native, SOS-inducible *uvrA* promoter (Supplementary Movie 1). We then assessed the ability of UvrA-YPet to execute NER in UV-survival assays. Compared with wild-type cells, *uvrA-YPet* cells exhibited somewhat poorer UV survival (Supplementary Fig. 1a). Considering that C-terminal fusions of UvrA are fully functional in NER^24,29^, this modestly lower survival of *uvrA-YPet* cells may be attributable to lower copy numbers.

Therefore, we measured the copy numbers of UvrA-YPet in *uvrA-YPet* cells grown in EZ-rich defined media supplemented with glucose (EZ-glucose) at 30 °C. Exponentially growing cells were deposited on a modified glass coverslip at the bottom of a flow cell and visualized by illumination with 514-nm laser light under continuous flow of growth medium (Fig. 2b). Images of *uvrA-YPet* cells revealed DNA-bound UvrA-YPet molecules that manifested as static foci and diffusive molecules contributing to cellular background fluorescence (Fig. 2c, Supplementary Movie 1). These observations are consistent with its role as a damage surveillance protein.

Exposure to laser light led to rapid loss of YPet signal due to photodarkening and photobleaching of the chromophore (Supplementary Movie 1). We used this loss of signal to measure copy numbers of UvrA-YPet in cells. Dividing the background-corrected cellular fluorescence intensity by the intensity of a single YPet molecule revealed a copy number of 16 ± 4 copies of UvrA-YPet per cell (Supplementary Fig. 1b–d). Copy numbers of UvrA are medium and condition dependent, ranging from 9–43 copies (minimal media) to 129 copies (rich media) per cell^30^. To confirm that the deficiencies in UV survival arise from lower UvrA-YPet copy numbers as opposed to a catalytically deficient protein, we created a low-copy plasmid (pUvrA-YPet, 3–4 copies/cell^31^) expressing UvrA-YPet under its native promoter. UvrA-YPet was expressed to the extent of 120 ± 28 copies per cell in Δ*uvrA/*pUvrA-YPet cells (Supplementary Fig. 1b–d) and fully complemented the Δ*uvrA* phenotype in UV-survival assays (Supplementary Fig. 1e). Therefore, we concluded that the copy numbers of UvrA-YPet expressed from the endogenous *uvrA* promoter represent lower copy numbers compared with untagged UvrA expressed in wild-type cells, likely reflecting a poorer efficiency of translation of the *uvrA-YPet* gene.

### Interval imaging strategy to measure DNA-binding kinetics

Continuous imaging of UvrA-YPet could not be used to measure DNA-binding lifetimes, since the loss of signal in a focus represents dissociation of bound UvrA-YPet from the site or photobleaching of the YPet in bound UvrA-YPet molecules. Consequently, measurement of interactions that last longer than the photobleaching lifetime is impossible. Instead we imaged UvrA-YPet using a strategy of performing time-lapse imaging with dark periods of varying intervals^32,33^ (for brevity, we term this mode of imaging interval imaging^23,25^) (Fig. 2d) that elegantly deconvolutes the lifetime of the interaction of UvrA-YPet with DNA and the lifetime of the fluorescent probe. Briefly, the introduction of a dark interval (*τ*_d_) between consecutive frames of duration (*τ*_int_) extends the observation time window. Here, the time between consecutive frames is denoted as time-lapse time (*τ*_tl_). By acquiring the same number of frames in each video collected with a different dark interval, the photobleaching rate (*k*_b_) is maintained constant (see Tables 1 and 2 and “Methods”), while the observation window is extended arbitrarily. From these videos, cumulative residence time distributions of DNA-bound reactivated UvrA-YPet are constructed. Since these distributions reflect a mixture of two populations (UvrA molecules that dissociate and YPet molecules that photobleached), fitting them to an exponential function yields an effective off rate (*k*_eff_)^25,32^. The product (*k*_eff_*τ*_tl_) is a linear function of true off rate (*k*_off_*τ*_tl_) and the normalized photobleaching rate (*k*_b_*τ*_int_)^25,32^. For purposes of illustration, the data can be represented as *k*_eff_τ_tl_ vs. τ_tl_ plots where the slope reveals the off rate *k*_off_ and the Y-intercept reveals *k*_b_. This interval imaging strategy enables accurate quantification of binding lifetimes over three orders of magnitude from 0.1 s to several minutes^23,25,32,34^.

**Table 1:** Summary of binding lifetimes of wild-type and mutant UvrA-YPet in the absence of exogenous DNA damage.

Strain (genotype)	Probe	Treatment	A_fast_ (%)	*τ*_fast_ (s)	A_slow_ (%)	*τ*_slow_ (s)	*k*_b_ (s^−1^)	Exponential fit	Figure
Δ*uvrA* Δ*uvrB* Δ*mfd*/pUvrA-YPet	UvrA-YPet (plasmid)	–	72 ± 2	1.6 ± 0.1	28 ± 2	24 ± 1	5.5 ± 0.1	2	3
*uvrA-YPet* Δ*uvrB* Δ*mfd*	UvrA-YPet (chr)	–	73 ± 4	1.4 ± 0.4	27 ± 4	22 ± 8	5.5 ± 0.5	2	3
Δ*uvrA/* pUvrA(Δ131–250)-YPet	UvrA(Δ131–250)-YPet (plasmid)	–			100	29.7 ± 0.8	5.2 ± 0.05	1	3
*uvrA-YPet* Δ*mfd*	UvrA-YPet (chr)	–	78 ± 2	1.5 ± 0.1	22 ± 2	8.7 ± 0.4	6.3 ± 0.2	2	3
*uvrA-YPet uvrB(*Δ*βHG)* Δ*mfd*	UvrA-YPet (chr)	–			100	148 ± 36	6.0 ± 0.2	1	3
*uvrA-YPet* Δ*uvrB*	UvrA-YPet (chr)	–	–	–	100	97 ± 18	7.3 ± 0.1	1	3
*uvrA-YPet*	UvrA-YPet (chr)	–	79 ± 2	1.9 ± 0.2	21 ± 2	12.0 ± 0.8	7.2 ± 0.2	2	4
*uvrA-YPet*	UvrA-YPet (chr)	rif	63 ± 3	1.5 ± 0.3	37 ± 3	9.6 ± 0.6	5.7 ± 0.2	2	4
Δ*uvrA*/pUvrA-YPet	UvrA-YPet (plasmid)	–	74 ± 2	2.0 ± 0.1	26 ± 2	19 ± 1	6.8 ± 0.1	2	4
Δ*uvrA*/pUvrA-YPet	UvrA-YPet (plasmid)	rif	75 ± 2	1.7 ± 0.1	25 ± 2	11.5 ± 0.6	7.3 ± 0.1	2	4

**Table 2:** Summary of binding lifetimes of UvrA-YPet and Mfd-YPet after UV exposure.

Strain (genotype)	Probe	Treatment	A_fast_ (%)	*τ*_fast_ (s)	A_slow_ (%)	*τ*_slow_ (s)	*k*_b_ (s^−1^)	Exponential fit	Figure
*uvrA-YPet* Δ*mfd*	UvrA-YPet (chr)	20 Jm^−2^ UVC (temporally averaged)	77 ± 3	1.6 ± 0.1	23 ± 3	13.1 ± 0.6	6.2 ± 0.2	2	5
*uvrA-YPet* Δ*mfd*	UvrA-YPet (chr)	20 Jm^−2^ UVC (0–25 min)	66 ± 1	1.6 ± 0.3	34 ± 1	9.6 ± 1	5.2 ± 0.3	2	5
*uvrA-YPet* Δ*mfd*	UvrA-YPet (chr)	20 Jm^−2^ UVC (25–50 min)	80 ± 5	1.3 ± 0.1	20 ± 5	13 ± 1	5.6 ± 0.4	2	5
*uvrA-YPet* Δ*mfd*	UvrA-YPet (chr)	20 Jm^−2^ UVC (50–75 min)	76 ± 5	1.9 ± 0.3	24 ± 5	15 ± 2	6.7 ± 0.3	2	5
*uvrA-YPet* Δ*mfd*	UvrA-YPet (chr)	20 Jm^−2^ UVC (75–100 min)	83 ± 3	1.5 ± 0.3	17 ± 3	15 ± 4	6.5 ± 0.5	2	5
*uvrA-YPet*	UvrA-YPet (chr)	20 Jm^−2^ UVC (temporally averaged)	74 ± 2	1.5 ± 0.1	26 ± 2	9.9 ± 0.4	5.5 ± 0.1	2	5
*uvrA-YPet*	UvrA-YPet (chr)	20 Jm^−2^ UVC (0–25 min)	71 ± 0.1	2.6 ± 0.7	29 ± 0.1	12 ± 3	4.8 ± 0.1	2	5
*uvrA-YPet*	UvrA-YPet (chr)	20 Jm^−2^ UVC (25–50 min)	70 ± 3	2.2 ± 0.4	30 ± 3	11.6 ± 0.9	5.9 ± 0.3	2	5
*uvrA-YPet*	UvrA-YPet (chr)	20 Jm^−2^ UVC (50–75 min)	78 ± 2	1.4 ± 0.1	22 ± 2	10.2 ± 0.6	5.6 ± 0.2	2	5
*uvrA-YPet*	UvrA-YPet (chr)	20 Jm^−2^ UVC (75–100 min)	87 ± 2	1.2 ± 0.2	13 ± 2	8.4 ± 0.6	5.8 ± 0.4	2	5
*mfd-YPet*	Mfd-YPet (chr)	20 Jm^−2^ UVC (temporally averaged)			100	12.0 ± 0.6	6.0 ± 0.1	1	5
*mfd-YPet*	Mfd-YPet (chr)	20 Jm^−2^ UVC (0–25 min)			100	13.7 ± 0.8	5.2 ± 0.2	1	5
*mfd-YPet*	Mfd-YPet (chr)	20 Jm^−2^ UVC (25–50 min)			100	11.5 ± 0.8	6.0 ± 0.2	1	5
*mfd-YPet*	Mfd-YPet (chr)	20 Jm^−2^ UVC (50–75 min)			100	10 ± 1	6.9 ± 0.2	1	5
*mfd-YPet*	Mfd-YPet (chr)	20 Jm^−2^ UVC (75–100 min)			100	8 ± 1	6.4 ± 0.3	1	5

### UvrA is long lived on DNA in the absence of UvrB and Mfd

**Fig. 3:**
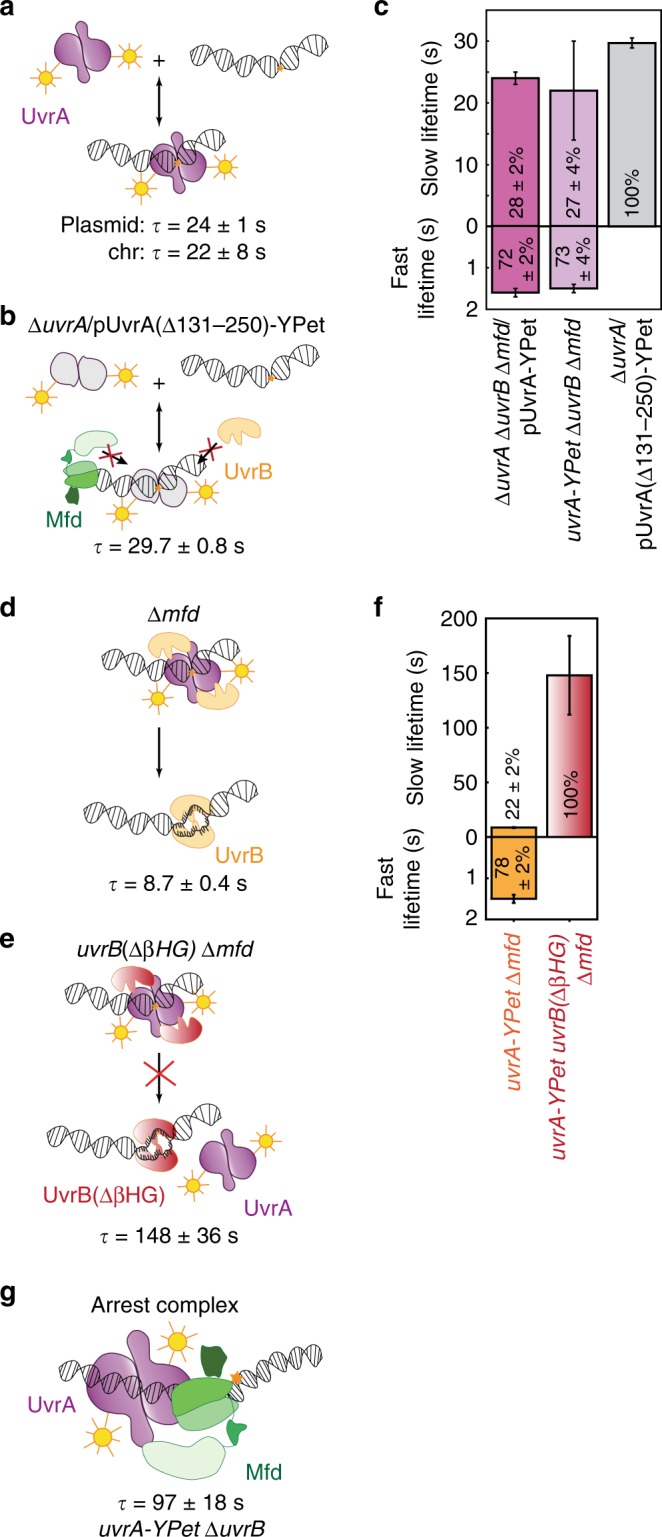
DNA-binding lifetimes of UvrA-YPet in the presence of UvrB. **a** Kinetics of UvrA-YPet interactions with DNA can be detected in the absence of UvrB and Mfd, in Δ*uvrA* Δ*uvrB* Δ*mfd* cells expressing UvrA-YPet from a low-copy plasmid or in Δ*uvrB* Δ*mfd* cells expressing UvrA-YPet from the chromosome. **b** Cartoon illustrates DNA binding by the mutant UvrA(Δ131–250)-YPet, which is defective in interacting with UvrB and Mfd. **c** Bar plots represent lifetimes of DNA-bound UvrA-YPet (plasmid: *n* = 34,927 counts from five repeats; chromosomal: *n* = 9703 counts from three repeats) and mutant UvrA(Δ131–250)-YPet (a total of *n* = 88,232 counts from four repeats) in the corresponding genetic background. Lifetimes were obtained from globally fitting the cumulative residence time distributions (CRTDs) (see also Supplementary Fig. 2a–f). Where two kinetic sub-populations are detected, the fast lifetime is displayed in the lower panel. Percentage represents the amplitude of kinetic sub-populations. **d** Cartoon illustrates loading of UvrB by UvrA. This may occur at sites of undamaged or damaged DNA. **e** Cartoon illustrates the complex formed by UvrA and the mutant UvrB(ΔβHG) that is deficient in loading reaction. **f** Bar plots represent lifetimes of DNA-bound UvrA-YPet in Δ*mfd* cells expressing either wild-type UvrB (*n* = 29,743 counts from 11 repeats) or mutant UvrB(ΔβHG) (*n* = 16,353 counts from two repeats). Lifetimes were obtained from globally fitting the CRTDs, with more than 1000 counts each CRTD (see Supplementary Fig. 2g–j). Where two kinetic sub-populations are detected, the fast lifetime is displayed in the lower panel. Percentage represents the amplitude of kinetic sub-populations. **g** Cartoon of the arrested Mfd-UvrA complex. See also Supplementary Fig. 3a, b. Error bars are standard deviations from ten bootstrapped CRTDs. Source data are provided as a Source Data file.

First, we interrogated UvrA binding kinetics in the absence of UvrB and Mfd in growing cells. To that end, we transformed cells lacking UvrA, UvrB and Mfd (Δ*uvrA* Δ*uvrB* Δ*mfd* cells) with pUvrA-YPet. In these cells, we expected that interactions of UvrA-YPet with DNA would reflect two of its key activities: binding to non-damaged DNA and binding to endogenous DNA damage produced as a by-product of cellular metabolism (Fig. 3a). Indeed, measurements of UvrA-YPet kinetics of dissociation in these cells revealed two populations with lifetimes that are an order of magnitude apart—a fast dissociating population with a lifetime (*τ*_UvrA|Δ*uvrA* Δ*uvrB* Δ*mfd*, fast_) of 1.6 ± 0.1 s (72 ± 2%) and a slow dissociating population with a lifetime (*τ*_UvrA|Δ*uvrA* Δ*uvrB* Δ*mfd*, slow_) of 24 ± 1 s (28 ± 2%) (summarized in Fig. 3c, Supplementary Fig. 2a, b, Table 1; error bars represent standard deviation of the bootstrap distribution of values obtained by performing global fits to CRTDs ten times). To eliminate the possibility that this measured lifetime is affected by cellular copy numbers of UvrA, we additionally created a strain that expresses *uvrA-YPet* from its endogenous promoter, and lacks the genes for *uvrB* and *mfd* (*uvrA-YPet* Δ*uvrB* Δ*mfd*). The measured lifetimes of UvrA-YPet in this strain were found to be identical within error (*τ*_UvrA|*uvrA-YPet* Δ*uvrB* Δ*mfd*, slow_ = 22 ± 8 s (27 ± 4%) and *τ*_UvrA|*uvrA-YPet* Δ*uvrB* Δ*mfd*, fast_ = 1.4 ± 0.4 s (73 ± 4%)) to those in the Δ*uvrA* Δ*uvrB* Δ*mfd*/pUvrA-YPet strain (Fig. 3a, c, Supplementary Fig. 2c, d and Table 1). Further, we also measured the binding lifetime of a mutant UvrA that is deficient in its interactions with UvrB and Mfd (Fig. 3b). Since UvrA interacts with both UvrB and Mfd via the interface formed by residues 131–250^35–37^, we expected that the labelled mutant UvrA lacking residues 131–250, UvrA(Δ131–250)-YPet, would be a faithful reporter of binding of kinetics of UvrA alone in *uvrB*^+^ *mfd*^+^ cells (Fig. 3b). Previous biochemical characterization of a UvrA(Δ131-248) mutant revealed that this mutant is unable to interact with UvrB and catalyze TCR and GGR^21^. Indeed, interval imaging of UvrA(Δ131–250)-YPet expressed from a low-copy plasmid (pUvrA(Δ131–250)-YPet) in Δ*uvrA* cells produced a single population with a binding lifetime (*τ*_UvrA(Δ131−250)_) of 29.7 ± 0.8 s (summarized in Fig. 3c, Supplementary Fig. 2e, f, Table 1). It is noteworthy that deletion of these residues leads to a complete abolishment of the short-lived species. The reasons for this loss may lie in structural differences between the wild-type and mutant proteins. Together, these results demonstrate that UvrA-YPet by itself is long lived on DNA. Notably, the lifetimes measured in our experiments reveal values that are larger than previous in vitro measurements of UvrA binding^29^. The presence of two binding lifetimes reveals the presence of two populations of UvrA on DNA.

### UvrB dissociates DNA-bound UvrA in cells lacking Mfd

Next, we studied the influence of UvrB on the DNA-binding lifetime of UvrA in cells lacking Mfd (*uvrA-YPet* Δ*mfd* cells; Fig. 3d) during normal growth. In these cells, UvrA-YPet dissociated with a short lifetime (*τ*_UvrA|Δ*mfd*, fast_) of 1.5 ± 0.1 s (amplitude: 78 ± 2%) and a long lifetime (*τ*_UvrA|Δ*mfd*, slow_) of 8.7 ± 0.4 s (22 ± 2%) (summarized in Fig. 3f, Supplementary Fig. 2g, h, Table 1). The lifetime of the slowly dissociating species observed in our measurements matches the lifetime detected for the dissociation of UvrA in the presence of UvrB previously (7 s)^29^, and is consistent with measurements of UvrB loading at sites of DNA damage^38^. Notably, the short lifetime is consistent with measurements from a previous study^24^; however, in this study a long-lived population of UvrA was not detected in the absence of exogenous DNA damage^24^.

In vitro studies have revealed that damage detection during NER proceeds via the loading of UvrB on DNA, followed by damage verification mediated via the helicase activity of UvrB^39–41^. To investigate whether the ability of UvrB to be loaded on DNA via its β-hairpin is essential for dissociation of UvrA from DNA, we measured the lifetime of UvrA-YPet in *uvrA-YPet* Δ*mfd* cells expressing the β-hairpin deletion mutant of UvrB from the native *uvrB* locus (“Methods”). This mutant, UvrB(ΔβHG), is inefficiently loaded on DNA in vitro^42^. The lifetime (*τ*_UvrA|*uvrB*(Δβ*HG*) Δ*mfd*_) of UvrA-YPet in cells expressing UvrB(ΔβHG) from the chromosome was 148 ± 36 s (100%); over 15-fold longer than that of UvrA-YPet in cells lacking Mfd (Fig. 3e, f, Supplementary Fig. 2i, j and Table 1). These data indicate that the UvrA-UvrB(ΔβHG) complex is arrested on DNA.

The lack of a short-lived species of UvrA in cells expressing mutant UvrB implies that the detectable population of UvrA can be sequestered to the chromosome in the form of a long-lived complex. Such a complex has been detected in single-molecule DNA stretching assays where UvrAB was demonstrated to slide on DNA^29^. In addition, we infer that in wild-type cells, loading of UvrB on DNA must promote the dissociation of UvrA. Indeed, our single-molecule live-cell imaging results highlight the physiological relevance of models constructed from in vitro studies that demonstrate that UvrB facilitates the dissociation of UvrA from DNA^42–44^. These findings lead us to suggest that the 8.7 s lifetime measured here corresponds to the lifetime of UvrA engaged in damage surveillance activities, wherein UvrA is turned over by UvrB loading.

### Mfd arrests UvrA on DNA in UvrB deficient cells

Next, we measured the lifetime of UvrA in cells lacking UvrB in the absence of exogenous DNA damage. Under these conditions, UvrA can form surveillance complexes (UvrA_2_) and interact with Mfd engaged with failed TECs. Interval imaging of UvrA-YPet in cells lacking UvrB (*uvrA-YPet* Δ*uvrB*) revealed a single long-lived UvrA species with a lifetime (*τ*_UvrA|Δ*uvrB*_) of 97 ± 18 s (Fig. 3g, Supplementary Fig. 3a, b). Since UvrA alone binds DNA with a lifetime of ~24 s, this highly stable species must reflect interactions with Mfd. We propose that this slowly dissociating species reflects the arrested Mfd-UvrA complex^12^ in cells lacking UvrB. Considering the copy numbers of UvrA measured here (16 per cell) and Mfd (22 per cell)^23^, and the reported picomolar affinity of UvrA for Mfd^12^, a large fraction of entire population of UvrA can be efficiently sequestered in complex with Mfd, consistent with the observation of only a single, slowly dissociating species in cells lacking *uvrB*. Our attempts at co-localization of UvrA and Mfd using the spectrally separated probes, YPet and PAmCherry were limited by the poor expression of Mfd-PAmCherry and UvrA-PAmCherry in cells under our standard growth conditions (Supplementary Note 1, Supplementary Fig. 3c, d).

### Mfd arrests UvrA on DNA in the absence of exogenous damage

We measured the residence time of DNA-bound UvrA in TCR-proficient cells in the absence of exogenous DNA damage. UvrA is recruited to DNA via Mfd to form the asymmetric handoff complex Mfd-UvrA_2_-UvrB that couples failed TECs to the repair machinery, unlike the symmetric UvrB-UvrA_2_-UvrB complex formed during damage surveillance in the absence of Mfd (Fig. 1). Since the residence time of Mfd is governed by UvrA during normal growth^23^, we anticipated three scenarios for the lifetime of UvrA in wild-type cells. First, if the residence time of UvrA-YPet is equal to 8.7 s, this would indicate either that the lifetimes of Mfd-UvrA and UvrA-UvrB interactions are similar, or that the lifetimes are different but the recruitment of UvrAB to RNAP-bound Mfd occurs so infrequently that only UvrAB complexes are detected. Second, a lifetime shorter than 8.7 s would suggest that Mfd promotes the dissociation of UvrA. Finally, a lifetime longer than 8.7 s would indicate that Mfd stabilizes UvrA.

**Fig. 4:**
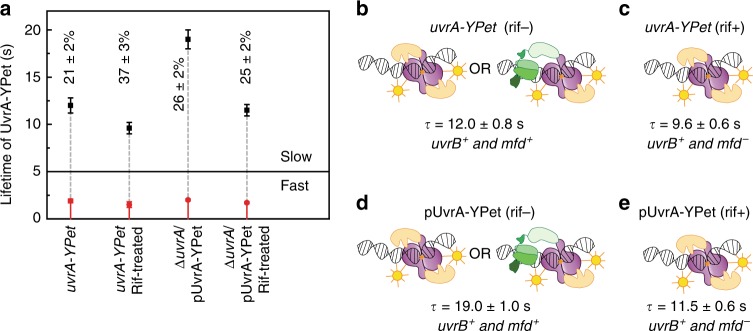
Dissociation kinetics of UvrA-YPet in TCR-competent cells. **a** Lifetimes of UvrA-YPet in *uvrA-YPet* cells or Δ*uvrA*/pUvrA-YPet cells untreated or treated with rifampicin. Lifetimes were obtained from globally fitting the CRTDs (see Supplementary Fig. 4a, c, e, g). Long lifetime is presented in black. Short lifetime is presented in red. Percentages represent the amplitude of the slowly dissociating population. **b** In the presence of UvrB and Mfd, UvrA-YPet in *uvrA-YPet* cells exhibited a long lifetime of 12.0 ± 0.8 s, reflecting UvrA interactions with UvrB and Mfd (*n* = 20,111 counts from eight repeats). **c** Rifampicin treatment abolishes Mfd-RNAP interactions, hence, UvrA-YPet is channelled towards interactions with UvrB, with the long lifetime found to be 9.6 ± 0.6 s (*n* = 15,355 counts from three repeats). **d** At eight-fold higher UvrA-YPet concentration obtained upon expression from the low-copy plasmid, the long lifetime of UvrA-YPet in Δ*uvrA*/pUvrA-YPet cells was found to be 19 ± 1 s, longer than that of UvrA-YPet in *uvrA-YPet* cells (12 s) (*n* = 19,853 counts from three repeats). **e** Upon rifampicin treatment, the long lifetime of UvrA-YPet in Δ*uvrA*/pUvrA-YPet cells reduced to 11.5 ± 0.6 s (*n* = 31,788 counts from four repeats). Error bars are standard deviations from ten bootstrapped CRTDs. Source data are provided as a Source Data file.

To distinguish between these three scenarios, we imaged UvrA-YPet in wild-type cells. Interval imaging of UvrA-YPet revealed a short-lived species with a lifetime (*τ*_UvrA, fast_) of 1.9 ± 0.2 s (79 ± 0.2%) and a long-lived species of UvrA with a lifetime (*τ*_UvrA, slow_) of 12.0 ± 0.8 s (21 ± 2%) (Fig. 4a, b, Supplementary Fig. 4a, b, Table 1). To identify the dependence of this lifetime on transcription, we imaged *uvrA-YPet* cells in conditions where Mfd-RNAP interactions are abolished by rifampicin (rif) treatment. Interval imaging of UvrA-YPet in rif-treated cells revealed a short-lived species with a lifetime (*τ*_UvrA|rif, fast_) of 1.5 ± 0.3 s (63 ± 3%) and a long-lived species of UvrA-YPet possessing a lifetime (*τ*_UvrA|rif, slow_) of 9.6 ± 0.6 s (37 ± 3%) (Fig. 4a, c, Supplementary Fig. 4c, d, Table 1). As expected, the long lifetime of UvrA-YPet in rif-treated cells matches that in cells lacking Mfd. Further, measurements of UvrA-YPet fluorescence in rif-treated cells did not reveal a loss of YPet fluorescence that might reflect enhanced degradation of UvrA-YPet upon rif treatment (Supplementary Fig. 4i). The decrease in the long-lived lifetime from 12.0 to 9.6 s indicates that a slowly dissociating, Mfd-bound species (lifetime greater than 8.7 s) of UvrA is lost upon rif treatment. These results lead us to conclude that the interactions of Mfd-UvrAB complexes are more stable on DNA than UvrAB complexes alone.

Since no mutants of UvrA have been identified that exclusively form the Mfd-UvrA_2_-UvrB complex but not the UvrA_2_UvrB_2_ complex in cells, measurement of the lifetime of UvrA in the Mfd-UvrA_2_-UvrB complex is difficult. Nevertheless, it is conceivable that the lifetime of the Mfd-UvrA_2_-UvrB complex could be influenced by the availability of downstream factors. We tested this hypothesis by measuring the lifetime of UvrA-YPet in wild-type cells expressing UvrA-YPet from the plasmid (Δ*uvrA*/pUvrA-YPet). Since UvrA can engage UvrB in solution, we reasoned that at higher expression levels, UvrA would bind UvrB to form the UvrA_2_B_2_ surveillance complex, leaving little free UvrB for engagement with Mfd-UvrA_2_ complexes. Consistent with this expectation, the data revealed a short-lived species with a lifetime (*τ*_UvrA|↑, fast_) of 2.0 ± 0.1 s (74 ± 2%) and a previously unencountered population of long-lived UvrA possessing a lifetime (*τ*_UvrA|↑, slow_) of 19 ± 1 s (26 ± 2%) (Fig. 4a, d, Supplementary Fig. 4e, f, Table 1).

In cells, UvrA is involved in target search (1.6 ± 0.1 s and 24 ± 1 s lifetimes, Fig. 3c) and damage surveillance as part of UvrA_2_B_2_ (8.7 s lifetime, Fig. 3f) in addition to Mfd-dependent UvrA(B) complexes (with lifetime of at least 12 s). To identify whether this long-lived UvrA species (19 ± 1 s lifetime) interacts with Mfd, we treated Δ*uvrA*/pUvrA-YPet cells with rif. Under this condition, we expected to recover the lifetime of UvrA as part of UvrA_2_ or UvrA_2_B_2_ complexes. Indeed, measurements of lifetimes of UvrA-YPet in rif-treated Δ*uvrA*/pUvrA-YPet cells revealed a lifetime (*τ*_UvrA|↑rif, slow_) of 11.5 ± 0.6 s (25 ± 2%) and a short lifetime (*τ*_UvrA|↑rif, fast_) of 1.7 ± 0.1 s (75 ± 2%) (Fig. 4a, e, Supplementary Fig. 4g, h, Table 1). The faster turnover of UvrA in response to rif treatment is consistent with the inference that the lifetime of UvrA in the Mfd-UvrA_2_-UvrB intermediate is longer than that in the UvrA_2_B_2_ intermediate in the absence of exogenous damage.

Notably, rif treatment of Δ*uvrA*/pUvrA-YPet cells yielded a lifetime (11.5 s) that is longer than that measured for rif-treated *uvrA-YPet* cells (9.6 s), and cells lacking *mfd* (8.7 s). The simplest explanation consistent with these observations is that under conditions of high relative UvrA/UvrB abundance the population is composed of UvrA_2_B_(2)_ complexes (lifetime of 8.7 s, Fig. 3d) and DNA-bound UvrA_2_ awaiting turnover by UvrB (lifetime of 24 s, Fig. 3a). We suggest that at higher cellular concentrations of UvrA relative to UvrB and Mfd, the existing population of UvrB is now required to turnover a greater number of UvrA molecules on undamaged DNA and at sites of endogenous damage, in addition to TCR intermediates. This model predicts that Mfd and UvrA must form a TCR intermediate whose disassembly is contingent on the arrival of UvrB. Together these results highlight two important features of prokaryotic NER: first, that damage surveillance by UvrA is highly dynamic and can be readily diverted from one pathway to the other depending on the condition, and second that the lifetimes of individual actors are determined by the presence of downstream factors.

### UvrA-Mfd interactions are prioritized in UV-irradiated cells

Next, we characterized the behaviour of UvrA in cells exposed to UV light. UV irradiation leads to the formation of photoproducts in cellular DNA^45^. These in turn elicit the induction of the SOS response during which the expression of NER factors is upregulated, permitting rapid removal of UV lesions from DNA^46–49^. To quantify this SOS-induced upregulation in real time, we monitored the relative abundance of UvrA in cells following UV irradiation.

**Fig. 5:**
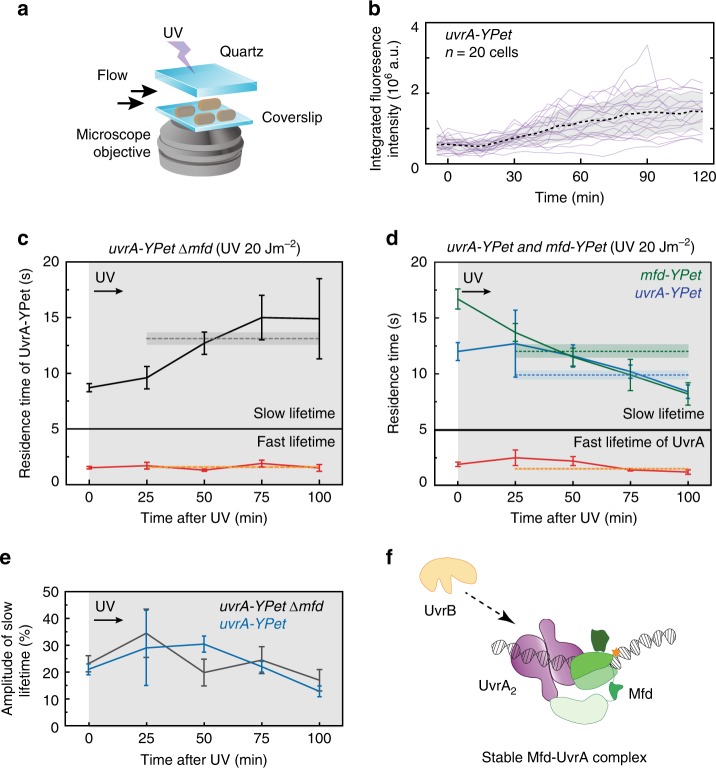
Lifetimes of DNA-bound UvrA and Mfd in response to UV irradiation. **a** Setup for in situ UV irradiation of cells followed by interval imaging at 30 °C for several hours after UV exposure. **b** Fluorescence intensity of single *uvrA-YPet* cells increases following exposure to UV light during the SOS response. **c** Lifetimes of UvrA-YPet in TCR-deficient cells as a function of time following UV exposure (see Supplementary Fig. 5a–d). The lifetimes at *t* = 0 min are reproduced from Fig. 4a. (After UV irradiation *uvrA-YPet* Δ*mfd* all data: *n* = 21,824 counts from four repeats; 0–25 min: *n* = 3243; 25–50 min: *n* = 5175; 50–75 min: *n* = 6079; 75–100 min: *n* = 5999 counts). **d** Lifetimes of UvrA-YPet in *uvrA-YPet* cells (long: blue, short: red) (after UV irradiation *uvrA-YPet* all data: *n* = 25,935 counts from four repeats; 0–25 min: *n* = 5359; 25–50 min: *n* = 8070; 50–75 min: *n* = 7481; 75–100 min: *n* = 4983 counts) or Mfd-YPet in *mfd-YPet* cells (green) (after UV irradiation *mfd-YPet* all data: *n* = 14,553 counts from four repeats; 0–25 min: *n* = 5133; 25–50 min: *n* = 3846; 50–75 min: *n* = 3119; 75–100 min: *n* = 2286 counts) as a function of time following UV exposure (Supplementary Fig. 6a–d). The lifetimes at *t* = 0 min are reproduced from Fig. 4a. Lifetime of Mfd-YPet at *t* = 0 min is reproduced from our previous work^23^. For **b** and **d**, lifetimes were obtained from globally fitting the CRTDs. Lifetimes of the fast and slowly dissociating sub-populations are shown in the lower and upper panels respectively. Dashed lines and error bands represent lifetimes and the corresponding standard deviations obtained from aggregated CRTDs within 100 min following UV exposure. Fitting results are available in Table 2. **e** Amplitudes of slowly dissociating species of UvrA-YPet in *mfd*^+^ (blue) or Δ*mfd* (black) cells carrying *uvrA-YPet*. See also Table 2 for fitting results. **f** UvrB (orange) controls the release of UvrA (purple) from UvrA-Mfd (green) intermediates. Error bars are standard deviations from ten bootstrapped CRTDs. Source data are provided as a Source Data file.

Time-lapse experiments on UV-irradiated *uvrA-YPet* cells allowed us to monitor the cellular fluorescence of tagged UvrA as a function of time. *uvrA-YPet* cells were immobilized in a flow cell with a quartz window and irradiated with 20 Jm^−2^ of damaging 254-nm UV light (Fig. 5a). This was followed by acquiring a single snapshot, every 5 min for 3 h. Quantification of cellular fluorescence intensities revealed that the integrated fluorescence intensities of single *uvrA-YPet* cells increase 30 min after UV exposure by threefold, consistent with the rapid deregulation of the SOS-inducible *uvrA* promoter (Fig. 5b)^47,48^ (see Supplementary Note 2).

We then measured the lifetime of UvrA in in situ UV-irradiated TCR-deficient cells (*uvrA-YPet* Δ*mfd*; 20 Jm^−2^ of 254 nm light) by performing interval imaging in four rounds, each lasting 25 min. Analysis of the complete data set revealed binding kinetics of UvrA-YPet corresponding to a short-lived species with a lifetime (*τ*_UvrA|Δ*mfd*, UV fast_) of 1.6 ± 0.1 s (77 ± 3%) and a long-lived species of UvrA corresponding to a lifetime of (*τ*_UvrA|Δ*mfd*, UV slow_) 13.1 ± 0.6 s (23 ± 3%) (Fig. 5c, Supplementary Fig. 5, Table 2). Strikingly the lifetime of the slowly dissociating species was larger than that measured in the absence of exogenous DNA damage (8.7 s, see Fig. 3d).

We wondered if the longer lifetime of UvrA detected in these experiments represented temporally averaged measurements. Since each set of interval measurements lasted 25 min, we proceeded to disaggregate each data set into the four constituent 25-min intervals after UV exposure. Analysis of the resulting data from each time window revealed that the measured lifetime of UvrA changes as a function of the experimental timeline after the UV pulse (Fig. 5c, Supplementary Fig. 5, Table 2). Indeed, in the first 25 min, the long lifetime of UvrA (9.6 ± 1 s) matched that measured in the absence of DNA damage (8.7 ± 0.4 s). This lifetime increased to a maximum of 15 ± 2 s in the 50–75 min time window, finally plateauing to 15 ± 4 s in the 75–100 min time window after UV exposure. There are two main takeaways from these experiments. First, the lifetime of short-lived UvrA does not change upon UV exposure, and is identical to that measured in the absence of any exogenous DNA damage. We conclude that this species is involved in binding undamaged DNA. Second, since the lifetime of long-lived UvrA changes upon UV exposure, we conclude that this species is engaged in DNA damage recognition.

Next, we repeated the interval imaging experiments on UV-irradiated wild-type cells (*uvrA-YPet*). In this case, UvrA-YPet exhibited two kinetic populations after UV exposure, a short-lived population with a lifetime of 1.5 ± 0.1 s (74 ± 2%) and a second, longer-lived population with a lifetime of 9.9 ± 0.4 s (26 ± 2%) (Fig. 5d, Supplementary Fig. 6, Table 2). As before, we disaggregated each data set into the four constituent 25-min intervals after UV exposure. In contrast to TCR-deficient cells, the measured lifetime of UvrA in wild-type cells remained low (8.4 ± 0.6 s) in the 75–100 min time window after UV exposure. These data indicate that UvrA is turned over faster in an Mfd-dependent manner during the SOS response.

Next, we plotted the amplitude of the long-lived species in each time window after UV irradiation (Fig. 5e). In *uvrA-YPet* cells, the fraction of the population dissociating with a slow off rate was greatest in the first 50 min after UV exposure (at ~30%), and subsequently dropped rapidly to 13 ± 2% at 100 min after UV. In comparison, *uvrA-YPet* Δ*mfd* cells exhibited a non-linear trajectory that nevertheless exhibited a similar reduction in the fraction of UvrA-YPet possessing a slow off rate. The most striking difference between the two strains was apparent at the 50 min time point—cells carrying *mfd* exhibited a 1.5-fold greater fraction of UvrA molecules dissociating with the slow lifetime. These results are broadly consistent with the expectation that as repair progresses, fewer unrepaired lesions are available for UvrA binding.

We followed these studies with an investigation of the binding lifetimes of Mfd. In the absence of exogenous DNA damage, the lifetime of Mfd is ~18 s^23^. Interval imaging of Mfd-YPet in *mfd-YPet* cells exposed to UV light revealed that the lifetime of Mfd-YPet dropped during the course of the SOS response, leading to an average lifetime (*τ*_Mfd|UV_) of 12 s (Fig. 5d, and Supplementary Fig. 7, Table 2). Strikingly, the binding lifetime of Mfd mirrored that of UvrA in wild-type cells after UV exposure, providing further evidence in support of an Mfd-UvrA complex in cells.

## Discussion

UvrA is the central player in NER since it performs critical functions in both GGR and TCR: first, it recognizes DNA damage as UvrA_2_ or UvrA_2_B_(2)_ and loads UvrB in GGR and second, it stimulates TCR by interacting with Mfd. In this work, we quantified the binding behaviour of fluorescently tagged UvrA in living cells over the experimentally accessible, and biologically relevant timescale of 0.1–1000 s.

UvrA exhibits two populations on DNA in untreated cells lacking UvrB and Mfd. The short-lived population possessing a lifetime of ~2 s is insensitive to rif or UV treatment, suggesting engagement in damage search. The long-lived population exhibiting a lifetime of 24 s is sensitive to the presence of UvrB, consistent with in vitro findings that UvrB promotes the dissociation of UvrA^42–44^. Using TCR-deficient strains and rif treatment, we assigned the lifetime of DNA-bound UvrA-UvrB interactions to be 8.7 s. This lifetime likely corresponds to loading of UvrB by UvrA on DNA since the loading-deficient UvrB mutant arrests UvrA for 148 s (see Fig. 3). Overall, our findings are consistent with a previous study that detected a short-lived UvrA species in untreated cells, and a long-lived species upon UV treatment^24^.

Direct detection of the Mfd-UvrA interaction in the background of UvrAB interactions is challenging. Nevertheless, four observations provide evidence for an Mfd-UvrA interaction in cells: first, UvrA-YPet foci in Δ*uvrB* cells are five-fold longer lived compared to Δ*mfd* Δ*uvrB* cells. Second, the long lifetime of UvrA in non-UV-treated cells is sensitive to rif treatment, leading to a loss of a slowly dissociating species of UvrA presumably engaged with Mfd. Third, in UV-treated cells, lifetimes of UvrA mirror those of Mfd. Finally, UvrA is turned over faster in an Mfd-dependent manner during the SOS response compared with untreated cells (Fig. 5). Together these observations suggest that Mfd and UvrA form a TCR handoff complex.

Curiously, the DNA-binding lifetimes of UvrA in Mfd-UvrA_2_-UvrB complexes are only distinguishable from those in UvrA_2_B_2_ under conditions where UvrB is present in limiting conditions, suggesting a role for UvrB in resolving Mfd-UvrA complexes in cells (Fig. 5f). A prediction of this hypothesis is that elevated concentrations of UvrB would lead to an enhanced turnover of Mfd in the Mfd-UvrA_2_-UvrB complex. The elevated copy numbers of UvrA and UvrB following UV treatment (Fig. 5b and refs. ^47,48^) may explain the faster turnover of UvrA during the SOS response.

The lifetime of UvrA in TCR-deficient UV-treated cells increases to 15 s, but remains low in UV-treated wild-type cells. An explanation for this striking difference may be found in work by Crowley and Hanawalt^46^. The differences observed in our experiments may fundamentally be attributable to the efficiencies with which UvrA recognizes cyclobutane pyrimidine dimers (CPDs) and 6–4 photoproducts. CPD lesions^50,51^, being less helix distorting than 6–4 photoproducts^52,53^, are inefficiently recognized by damage recognition factors in NER across organisms^54,55^. CPD recognition by RNAP and subsequent TCR at such sites is a major mechanism for CPD removal after UV treatment^46^. Compared with untreated cells that remove greater than 80% of CPD lesions within 40 min following UV treatment, rif-treated cells removed less than 50% of CPD lesions at the same time point^46^. In contrast, 6–4 photoproduct removal was uninfluenced by rif treatment^46^. Further, this report also demonstrated that the copy number of UvrAB determines whether CPDs are efficiently removed following UV treatment. Considering the low expression level of UvrA-YPet in our strain (16 ± 4 monomers per cell during normal growth), the initial time window in which UvrA is turned over rapidly in Δ*mfd* cells may reflect repair of 6–4 photoproducts. The later time windows in which UvrA exhibits a lifetime of 15 s may reflect repair of CPD lesions that are poorly detected by UvrA. Extending this argument, the prediction from the Crowley and Hanawalt study is that the rapid turnover of UvrA in wild-type cells in the 25–100 min time window reflects the repair of CPDs via TCR. Indeed, we found that the binding lifetimes of Mfd mirror those of UvrA in this time period. Further, this finding is consistent with previously established biphasic repair of UV lesions—rapid removal of 6–4 photoproducts is followed by a slower repair of CPDs^46^.

Finally, our data suggest that the enhancement of the rate of repair in TCR vs. GGR measured in bulk is best explained by enhanced target search in TCR compared with GGR. This model has been previously proposed in the literature based on evidence from in vitro studies^6,12^. In this model, stalled RNAP is recognized by Mfd, leading to the exposure of Mfd’s UvrB-homology module that in turn acts as a flag for UvrAB. Damage recognition by UvrAB would then follow initial recruitment to the site of the lesion. In contrast, target search during global genomic repair would comprise of repeated cycles of three-dimensional diffusion of UvrA(B) to sites of undamaged DNA followed by subsequent turnover of UvrA by UvrB until the damage surveillance complex stochastically encounters damaged DNA.

## Methods

### Construction of strains

Wild-type MG1655 was a generous gift from the Cox lab (UW Madison). *Escherichia coli* MG1655 *uvrA-YPet* was constructed using the Datsenko–Wanner protocol^28^ for λ Red recombination using the linker sequence (SAGSAAGSGEF)^23^. Briefly, the *uvrA-YPet* strain was constructed as follows: first, the YPet gene from *pYpet* (see Supplementary Table 4 for sequence) was amplified using UvrA_YPet_fw (TCGCG GAGTG CGAAG CATCA CACAC GGCAC GCTTCCTTAA GCCGA TGCTG TCGGCTGGCTCCGCTGCTGGTTCTGGCGAATTC ATG TCT AAA GGT GAA GAA TTA TTC ACT GGT G) and UvrA_rev primers (GCTGG TGCAA CTCTG AAAGG AAAAG GCCGC TCAGA AAGCG GCCTT AACGA GAA GTT CCT ATT CTC TAG AAA GTA TAG) using the high-fidelity KOD polymerase (Toyoba Global) with annealing temperature of 70 °C. Deletion constructs (*uvrA::KanR*, *uvrB::KanR* and *mfd::KanR*) were created by replacing the indicated gene with a kanamycin cassette flanked by FRT sites^23^. Briefly, Δ*uvrA* cells were created by applying the Datsenko–Wanner protocol using the UvrA_del_507_fw primer (CGGTA GCACC ATGCC ACCGG GCAAA AAAGC GTTTA ATCCG GGAAA GGTGA ccctttcgtcttcaagaattc) and UvrA_del_507_rev (GCTGG TGCAA CTCTG AAAGG AAAAG GCCGC TCAGA AAGCG GCCTT AACGA ggccacgatgcgtccggcgta) that prime the pEAW507 plasmid (see Supplementary Table 4) to create a FRT-Kan-FRT cassette carrying flanking sequences that are homologous to the sequences on either side of the *uvrA* gene. Δ*uvrB* strains were similarly constructed using the UvrB_del_507_fw (GACGA GTAAA ATTAC ATACC TGCCC GCCCA ACTCC TTCAG GTAGC GACTC ccctttcgtcttcaagaattc) and UvrB_del_507_rev (TAGCG CATCA GGCTG TTTTC CGTTT GTCAT CAGTC TTCTT CGCTA TCCTG ggccacgatgcgtccggcgta) primers. In each case, wild-type electrocompetent MG1655 cells (carrying the temperature sensitive, amp^R^ pKD46 plasmid, and induced with L-arabinose) were transformed with 50 ng of gel purified PCR amplicons (QIAquick Gel extraction kit, Qiagen) and plated on Luria Bertani (LB) agar plates containing kanamycin (40 μg/mL) incubated at 37 °C overnight. Colonies obtained in the kanamycin plates were then transferred to two plates each containing either ampicillin (100 μg/mL) or kanamycin (40 μg/mL). Colonies that grew on kanamycin plates but not on ampicillin plates were then checked for the presence of YPet (or loss of the *uvrA* or *uvrB* gene) using sequencing primers (see Supplementary Table 2 for sequences). Candidate colonies were further 16-streaked on kanamycin plate (40 μg/mL) to obtain single colonies, which were then sequenced. Positive colonies with the correct sequence were stored as DMSO freezer stocks at −80 °C. An additional set of strains was created wherein the kanamycin cassette was removed using pLH29 that expresses the FLP recombinase. P1 transduction was then used to create strains expressing *uvrA-YPet* in the indicated deletion backgrounds.

Derivatives of *uvrA-YPet* expressing mutant UvrB(ΔβHG) from the chromosome were constructed using scar-less CRISPR-Cas9 assisted λ Red recombination as previously described^56,57^. Briefly, *uvrA-YPet* electrocompetent cells were transformed with pKD46^28^ and pCas9^56^ and selected on LB plates containing ampicillin (50 μg per mL) and chloramphenicol (25 μg per mL) following incubation at 30 °C overnight. Since pCas9 is prone to recombination events^56^, the resulting colonies were screened with colony PCR to confirm the presence of the full-length Cas9 gene, using primers (Supplementary Table 2) targeting upstream and downstream of the Cas9 gene in pCas9.

Next, *uvrA-YPet* cells harbouring pKD46 and pCas9 were made electrocompetent. First, the cells were grown at 30 °C with shaking at 200 rpm in 50 mL of LB containing ampicillin (50 μg per mL) and chloramphenicol (25 μg per mL). In these cells, Cas9 was constitutively expressed. The expression of λ Red recombination proteins was induced with 0.2% L-arabinose (w/v) when cells reached a density around 0.4 (OD_600_). When the optical density reached 0.8, cells were harvested at 4 °C and washed twice with ice-cold water, with an additional wash using 10% glycerol to obtain electrocompetent cells. Finally, aliquots containing 40 μL of cells in microcentrifuge tubes were snap-frozen in liquid nitrogen and stored at −80 °C.

Cas9 endonuclease activity was targeted to the vicinity of the desired point mutations on the *E. coli* chromosome with the help of guide RNAs expressed from pCRISPR-UvrB-Y96A (Supplementary Table 3), which we created following protocols from ref. ^56^. The point mutations were introduced by recombining the foreign ssDNA (Supplementary Table 2) into the chromosome. The ssDNAs were 80- to 90-nt oligos flanked by 40-nt of sequence homologous to the *E. coli* chromosome on either sides of the desired mutation. Base changes were selected to be within five bases from the PAM sequence.

Aliquots of cells expressing Cas9 and λ Red recombination proteins were transformed with 30 ng of pCRISRP-UvrB-Y96A plasmid and 500 ng of ssDNA. Positive colonies were selected on LB plates containing 50 μg per mL of kanamycin and 25 μg per mL of chloramphenicol at 37 °C, and were screened by colony PCR, and the promoter and gene sequences were sequence verified. pCRISPR-UvrB-Y96A was cured by propagating cells on LB plates for a week at 42 °C. This is critical for subsequent rounds of P1 transduction to create *uvrA-YPet uvrB(*Δβ*HG)* Δ*mfd*. Additional information on the cell lines can be found in Supplementary Table 1.

### Construction of plasmids used in this study

pUvrA-Ypet was constructed by first amplifying the *uvrA* gene from MG1655 using UvrA_FWc primer (TGG GTA CCG GGC CCG CGA TTG TAC CAT TAC CAA TAG) and UvrAYPET_revc (AGC CAG CCG Aag cga tcg cCA GCA TCG GCT TAA GGA) using Phusion High-Fidelity DNA polymerase (New England Biolabs) and an annealing temperature of 63 °C. The insert thus generated was PCR purified (QIAquick PCR Purification Kit, Qiagen) and digested using *Apa*I/*Xba*I. The pHH001 backbone^23^ (carrying a spectinomycin marker) digested using *Apa*I/*Xba*I and additionally with *Xho*I to digest the released Mfd fragment first at 25 °C for 2 h, followed by an additional 2 h at 37 °C. Following this, standard molecular biology techniques were employed to ligate the digested insert into the vector, followed by selection on spectinomycin plates. Colonies were then screened for the presence of the insert, followed by plasmid isolation (QIAprep Spin Miniprep Kit, Qiagen). Isolated plasmids were sequence verified to obtain pUvrA-YPet. The UvrA(Δ131–250) sequence was synthesized by IDT (United States) and received as an insert in the standard backbone IDT vector (amp^R^). This insert was PCR amplified using UvrA_FWc and the reverse primer RecASZ2_rev (GTGGCGGCCGCTCTA) using Phusion polymerase (annealing temperature of 65 °C) and visualized in a 1% agarose gel to obtain a single band of the expected size. Following PCR cleanup (QIAquick PCR Purification Kit, Qiagen), the insert was then digested with *Apa*I/*Asis*I (4 h at 25 °C followed by 4 h at 37 °C) followed by further purification (Qiagen, PCR purification kit). In parallel, pHH002 (low-copy vector that expresses Mfd(L499R) under its native promoter cloned between *Apa*I/*Asis*I^23^) was digested to release the fragment containing the *mfd(L499R)* gene. The PCR amplified mutant *uvrA* sequence was thus sub-cloned into pHH002 using standard molecular biology protocols. Successful transformants following ligation were screened for the presence of the insert, followed by sequencing to obtain pUvrA(Δ131–250)-YPet. Plasmids were sequenced on both strands prior to use.

### Cell culture for imaging

Cells were imaged in quartz-top flow cells as described previously^23^. Briefly, flow cells were assembled using a clean quartz piece (Proscitech, Australia) and a bottom cleaned and (3-Aminopropyl)triethoxysilane (Alfa Aesar, A10668, UK)-treated coverslip (Marienfeld, Deckglaser, 24 mm × 50 mm, No. 1.5, German) using double-sided sticky tape (970XL ½ × 36 yd, 3 M, United States), and sealed with 5-min epoxy (Parfix). Quartz top pieces were designed to be able to insert inlet and outlet tubing (PE-60, Instech Labs). Prior to imaging, cells were revived from a −80 °C DMSO stock in 500 μL of EZ-rich defined media (Teknova, CA, US), supplemented with 0.2% (v/v) glucose in 2-mL microcentrifuge tubes at 30 °C. Cultures were set to shake in an Eppendorf Thermomixer C (Eppendorf, Australia) at 1000 rpm. On the following day, cultures were reset by inoculating fresh growth medium 1:200 fold, and continued to shake for ~3 h at 30 °C prior to imaging. For experiments involving plasmid-expressed UvrA-YPet or UvrA(Δ131–250)-YPet, spectinomycin (50 μg per mL) was added to the growth media. Cells in early exponential phase were loaded in flow cells at 30 °C, followed by a constant supply of aerated EZ-rich defined media at a rate of 30 µL per min, using a syringe pump (Adelab Scientific, Australia).

### Single-molecule live-cell imaging

Single-molecule fluorescence imaging was carried out with a custom-built microscope as previously described^23^. Briefly, the microscope comprised a Nikon Eclipse Ti body, a 1.49 NA 100× objective, a 514-nm Sapphire LP laser (Coherent) operating at a power density of 71 W cm^−2^, an ET535/30m emission filter (Chroma) and a 512 × 512 pixel^2^ EM-CCD camera (either Photometrics Evolve or Andor iXon 897). The microscope was operated in near-TIRF illumination^58^ and was controlled using NIS-Elements (Nikon). PAmCherry-tagged proteins were imaged as described previously^23^.

Fluorescence images were acquired in time-series format with 100 ms frames. Each video acquisition contained two phases. The first phase (50 frames) aimed to lower background signal by continuous illuminating, causing most of the fluorophores to photo-bleach or to assume a dark state. The second phase (single-molecule phase lasting for 100 frames) is when single molecules can be reliably tracked on a low background signal. In the second phase, consecutive frames were acquired continuously or with a delay time (*τ*_d_).

### Image analysis

Image analysis was performed in Fiji^59^, using the single-molecule biophysics plugins (available at https://github.com/SingleMolecule/smb-plugins), and MATLAB. First, raw data were converted to TIF format, following by background correction and image flattening as previously described^23^. Next, foci were detected in the reactivation phase by applying a discoidal average filter (inner radius of 1 pixel, outer radius of 3 pixels), then selecting pixels above the intensity threshold. Foci detected within 3-pixel radius (318 nm) in consecutive frames were considered to belong to the same binding event.

### Interval imaging for dissociation kinetics measurements

Interval imaging was performed as described previously^23,25^. Briefly, the photobleaching phase contained 50 continuous 0.1-s frames. In phase II, 100 discontinuous 0.1-s frames (*τ*_int_ = 0.1 s) were collected with time-lapse time (*τ*_tl_) ranging from *τ*_tl_ = (0.1, 0.2, 0.5, 1, 2, 4, 8, 10 s). In each experiment, videos with varying *τ*_d_ were acquired. Foci were detected using a relative intensity threshold of 7 or 8 above the background as appropriate. Depending on the construct being imaged, between 3 and 15 repeats of each experiment were collected for each strain. Cumulative residence time distribution of binding events detected in all data sets were generated for each *τ*_tl_. Cumulative residence time distributions were global fit to single- or double-exponential models to obtain binding lifetimes and amplitudes of kinetic sub-populations (custom-written MATLAB codes^25^). Here, the choice of fitting model was decided based on the shape of *k*_eff_*τ*_tl_ vs. *τ*_tl_ plot (ref. ^32^ and guidelines from ref. ^25^), in which the effective decay rate *k*_eff_ represents the sum of the normalized photobleaching rate *k*_b_ and the off rate *k*_off_ (*k*_eff_ = *k*_b_*τ*_int_ *τ*_tl_^−1^ + *k*_off_). The linearity of *k*_eff_*τ*_tl_ vs. *τ*_tl_ plot represents a single kinetic population, whereas deviations from linear fits indicate multiple kinetic sub-populations. The corresponding *k*_eff_τ_tl_ vs. *τ*_tl_ plot was obtained as described previously^23^, with the shaded error bar representing standard deviations of ten bootstrapped samples deriving from 80% of the compiled binding events (custom-written MATLAB codes). Given that this method is based on fitting mixtures of exponential models to the data, this approach reliably resolves off rates that are at least threefold apart. In experiments involving rif treatment, cells were incubated in growth media containing rif (50 μg per mL) for 30 min in the flow cell prior to imaging.

### Experiments involving UV irradiation

UV survival of wild-type, *uvrA-YPet* and Δ*uvrA* cells (Supplementary Fig. 1a) was assayed by first growing these strains in LB to an OD_600_ of ∼0.6. Following this 4 × 10^7^ cells were harvested and resuspended in 200 μL of 100 mM MgSO_4_ after two rounds of washing in 200 μL of 100 mM MgSO_4_. Next, 20 μL of the cell suspension were sandwiched between a UV-transparent quartz piece and a glass slide followed by exposure to 0, 5, 10, 20 or 40 Jm^−2^ of 254 nm UV light delivered by a short-wave UV lamp (Herolab, Germany). Following UV irradiation, 5 μL of the cell suspension were taken and serially diluted tenfold, five times. Finally, 50 μL of the 10^5^ dilution were plated in triplicate on LB plates. Plates were then incubated overnight at 37 °C, and colony forming units were counted the next day. Surviving fraction was calculated as the ratio of colonies observed in the irradiated condition to those observed in the un-irradiated condition. Experiments were repeated with two biological repeats, and three technical repeats for each strain and each condition. Plate reader-based UV-survival assays were performed as described previously in refs. ^23,60^. Briefly, cells were grown in LB medium to an OD_600_ between 0.6 and 1 and resuspended in 100 mM MgSO_4_ following two washes in MgSO_4_. A 20 μL drop was placed on a quartz plate. UV irradiation (0, 10, 20 or 40 Jm^−2^) was provided as before using a short-wave UV lamp (Herolab, German). Five microliters of the cell suspension were recovered and diluted tenfold, five times in LB. For each condition, sample was plated in triplicate in a flat-bottom 96-well plate (Costar). In addition six wells contained only LB medium to serve as blanks. Data presented reflect mean and standard error of the mean from two biological repeats, each performed in triplicate. UV irradiation in microscopy experiments was delivered in situ as described previously^60^. The UV flux was measured prior to UV irradiation in all experiments, and the exposure time was adjusted to provide a dose of 20 Jm^−2^. For experiments involving interval imaging following UV exposure, *τ*_tl_ = (0.1, 0.3, 1, 3, 10 s) values were used to minimize the time taken to complete one round of interval imaging.

### Reporting summary

Further information on research design is available in the Nature Research Reporting Summary linked to this article.

## Supplementary information

Supplementary Information

Description of Additional Supplementary Files

Supplementary Movie 1

Reporting Summary

## Data Availability

Data supporting the findings of this manuscript are available from the corresponding author upon reasonable request. A reporting summary for this Article is available as a Supplementary Information file. All source data underlying Figs. 2c, d, 3 and 4c–e and Supplementary Figs. 1–7 are provided as a Source Data file.

## Source Data

Source Data

## Code availability

Custom code used for data analyses has been made publicly available at github.com/hghodke/bacterial_live_cell_interval_imaging.

Image analysis was performed in Fiji^59^, using the single-molecule biophysics plugins (available at: https://github.com/SingleMolecule/smb-plugins).
