# Supplementary material for: Single-molecule live-cell imaging visualizes parallel pathways of prokaryotic nucleotide excision repair
Source: Nat Commun. 2020 Mar 20;11:1477. doi: 10.1038/s41467-020-15179-y (PMC7083872; doi:10.1038/s41467-020-15179-y)
Supplement: Supplementary file 1 — Supplementary Information [file 41467_2020_15179_MOESM1_ESM.pdf]

Supplementary Information file

Single-molecule live-cell imaging visualizes parallel pathways of prokaryotic nucleotide excision repair

Harshad Ghodke[1,2], Han Ngoc Ho[1,2], Antoine M van Oijen[1,2]

[1]Molecular Horizons and School of Chemistry and Molecular Bioscience, University of Wollongong, Wollongong, New South Wales, 2522, Australia

[2] Illawarra Health and Medical Research Institute, Wollongong, New South Wales, 2522, Australia

Correspondence and requests for materials should be addressed to H.G. (email: harshad@uow.edu.au)

This PDF file includes:

Supplementary Figures 1 - 7
Supplementary Tables 1 - 4
Supplementary Notes 1 - 2
Supplementary References

Supplementary Figures

Supplementary Figure 1:

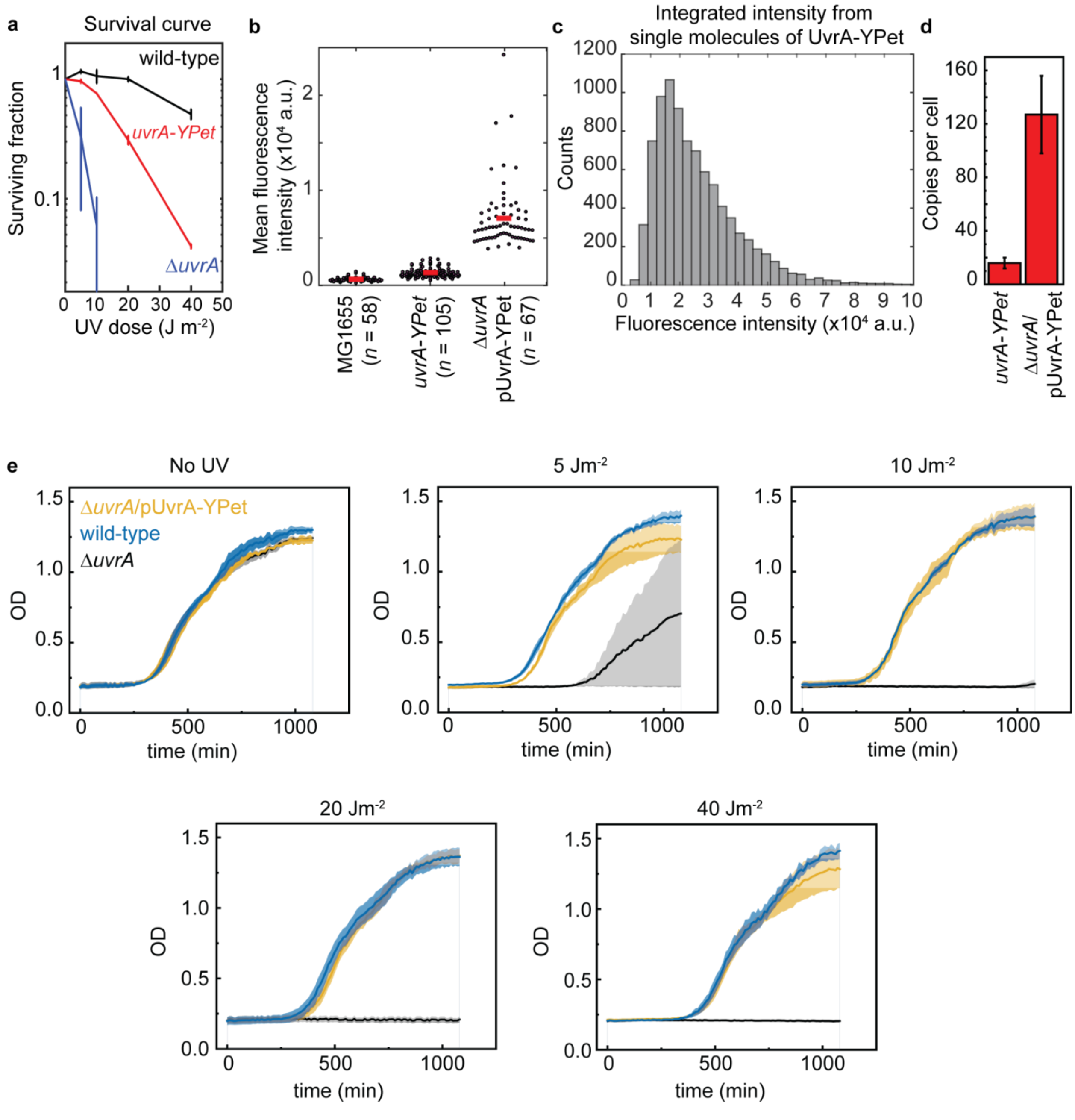

Supplementary Figure 1:
a. Survival assay showing survival of wild-type (black), *uvrA-YPet* (red) and Δ*uvrA* (blue) cells upon exposure to 20 Jm^{-2} of 254-nm UV light. Error bars represent standard error of the mean from two independent experiments, each experiment involved three technical replicates.

b. Mean fluorescence intensity of wild-type MG1655, *uvrA-YPet* cells and cells carrying a low-copy plasmid expressing UvrA-YPet upon excitation with 514-nm light. Red line indicates mean of the distribution. *n*, number of cells.

c. Histogram of integrated fluorescence intensities of single UvrA-YPet foci in *uvrA-YPet* cells upon excitation with 514-nm light.

d. Bar plots show mean copy number of UvrA-YPet measured in *uvrA-YPet* cells (*n* = 105 cells) and Δ*uvrA*/pUvrA-YPet cells (*n* = 67 cells). Error bars are standard deviations.

e. Growth curves (OD 600 nm; LB medium 30°C in a 96-well plate) of UV irradiated (0, 5, 10, 20 and 40 Jm^{-2} of UVC radiation) of the Δ*uvrA*/pUvrA-YPet strain (yellow line) compared to Δ*uvrA*/pJM1071 (empty vector; black line) and wild-type cells carrying the pJM1071 vector (blue line) reveal that UvrA-YPet expressed from the plasmid complements a Δ*uvrA* phenotype. Solid lines represent average of two biological replicates, each performed in triplicate. Shaded areas represent standard error of mean of two biological replicates.

Source data are provided as a Source Data file.

Supplementary Figure 2:

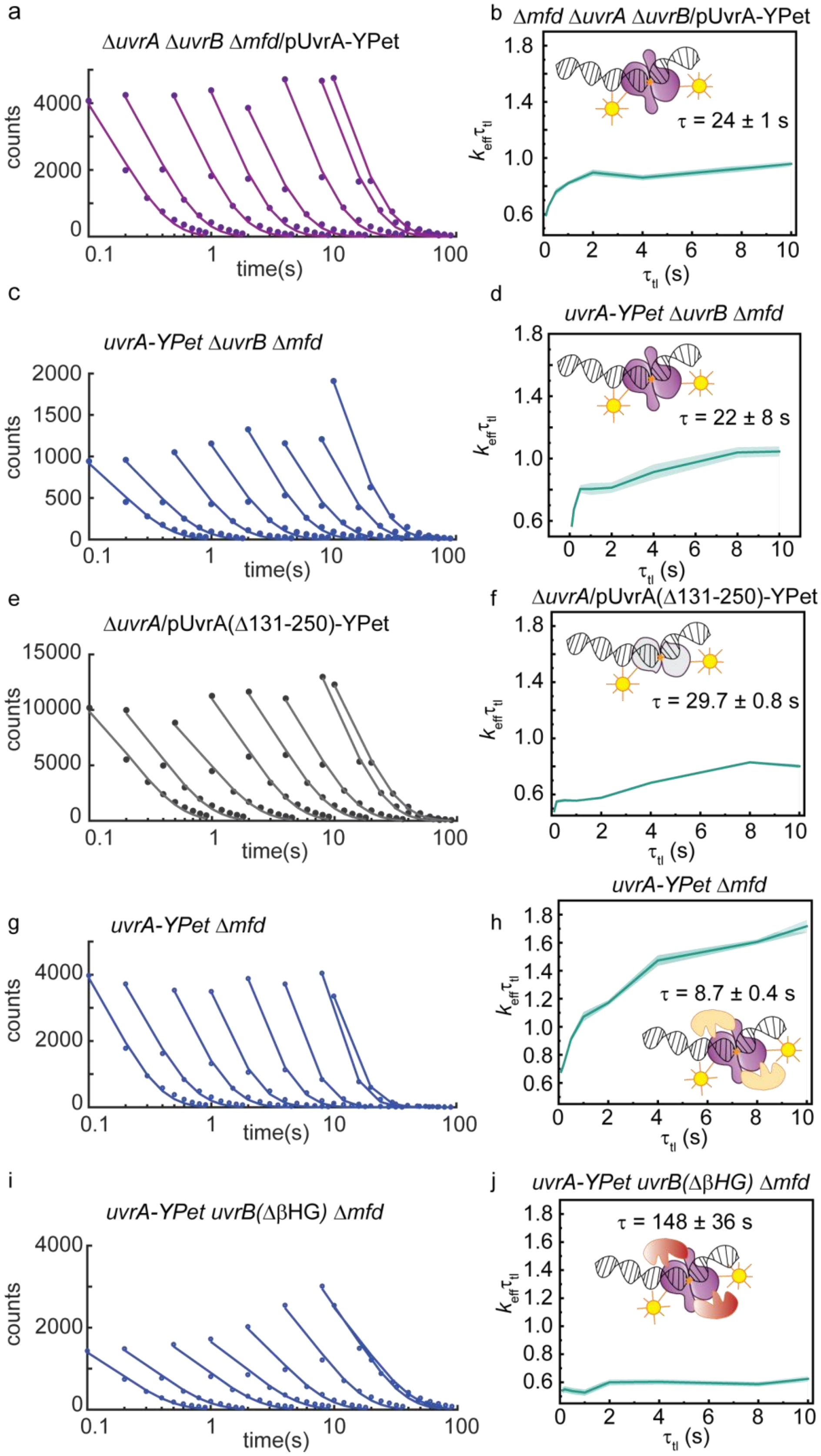

Supplementary Figure 2:
a. Cumulative residence time distributions (CRTDs, circles) obtained from interval imaging of UvrA-YPet in Δ*uvrA* Δ*uvrB* Δ*mfd* cells. Lines are mono-exponential fits to CRTDs.

b. The $k_{eff}\tau_{tl}$ plot obtained from fitting CRTDs of UvrA-YPet in Δ*uvrA* Δ*uvrB* Δ*mfd* cells. Shaded error bands are standard deviations from ten bootstrapped samples. Cartoon (inset) illustrates UvrA-YPet (purple) in complex with DNA.

c. Cumulative residence time distributions (CRTDs, circles) obtained from interval imaging of UvrA-YPet in *uvrA-YPet* Δ*uvrB* Δ*mfd* cells. Lines are mono-exponential fits to CRTDs.

d. The $k_{eff}\tau_{tl}$ plot obtained from fitting CRTDs of UvrA-YPet in *uvrA-YPet* Δ*uvrB* Δ*mfd* cells. Shaded error bands are standard deviations from ten bootstrapped samples. Cartoon (inset) illustrates UvrA-YPet (purple) in complex with DNA.

e. CRTDs (circles) obtained from interval imaging of UvrA(Δ131-250)-YPet in Δ*uvrA* cells. Lines are mono-exponential fits to CRTDs.

f. The $k_{eff}\tau_{tl}$ plot obtained from fitting CRTDs of UvrA(Δ131-250)-YPet in Δ*uvrA* cells. Shaded error bands are standard deviations from ten bootstrapped samples. Cartoon (inset) illustrates UvrA(Δ131-250)-YPet (grey) in complex with DNA.

g. CRTDs (circles) obtained from interval imaging of UvrA-YPet in *uvrA-YPet* Δ*mfd* cells. Lines are mono-exponential fits to CRTDs.

h. The $k_{eff}\tau_{tl}$ plot obtained from fitting CRTDs of UvrA-YPet in *uvrA-YPet* Δ*mfd* cells. Shaded error bands are standard deviations from ten bootstrapped samples. Cartoon (inset) illustrates the complex formed by UvrA-YPet (purple) and UvrB (orange) with DNA.

i. CRTDs (circles) obtained from interval imaging of UvrA-YPet in *uvrA-YPet uvrB(*ΔβHG*)* Δ*mfd* cells. Lines are mono-exponential fits to CRTDs.

j. The $k_{eff}\tau_{tl}$ plot obtained from fitting CRTDs of UvrA-YPet in *uvrA-YPet uvrB(*ΔβHG*)* Δ*mfd* cells. Shaded error bands are standard deviations from ten bootstrapped samples. Cartoon (inset) illustrates the complex formed by UvrA-YPet (purple) and UvrB(ΔβHG) (red) with DNA.
Source data are provided as a Source Data file.

Supplementary Figure 3:

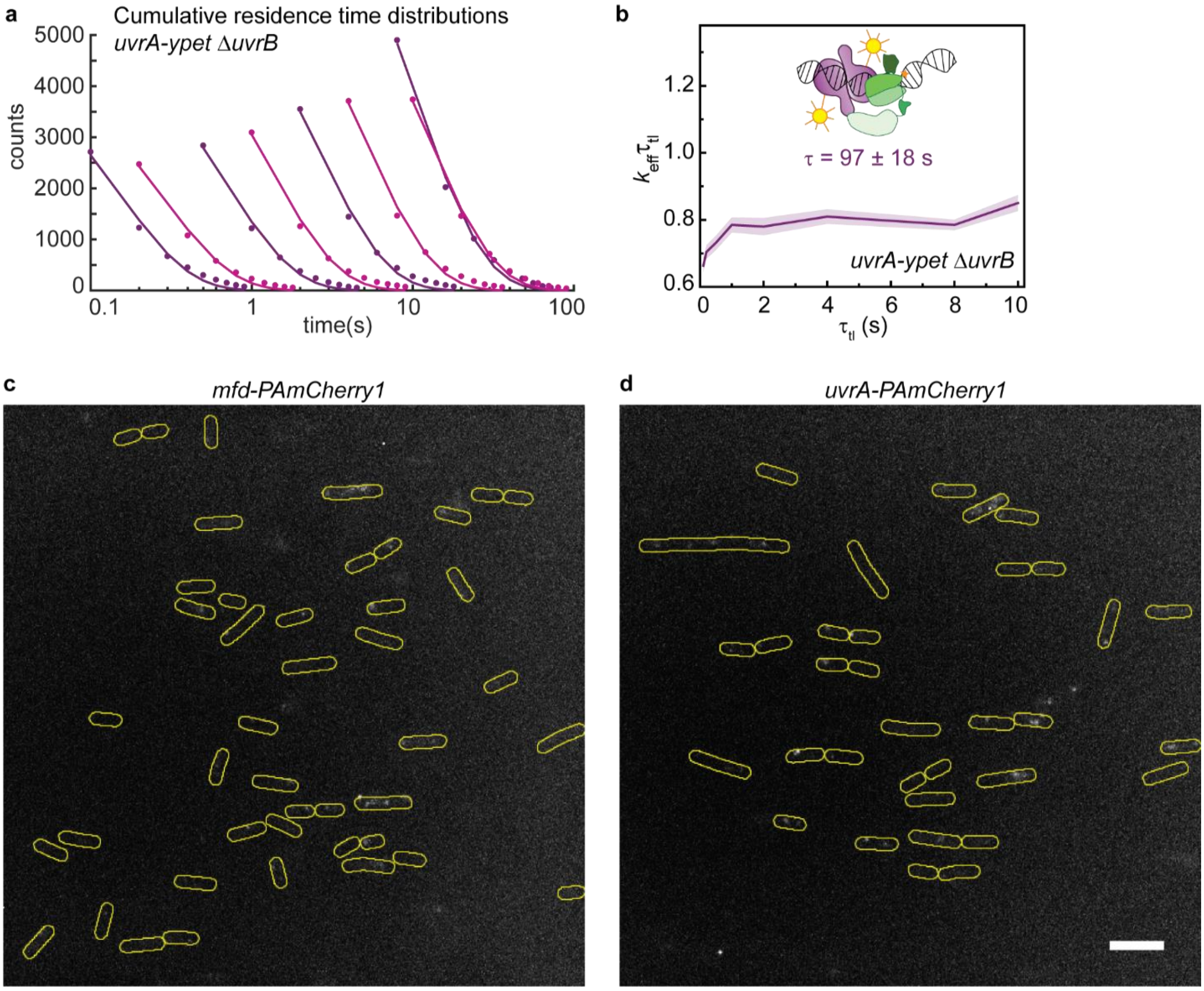

Supplementary Figure 3:
a. CRTDs (circles) obtained from interval imaging of UvrA-YPet in *uvrA-YPet* Δ*uvrB* cells. Lines are mono-exponential fits to CRTDs.

b. The $k_{eff}\,\tau_{tl}$ plot obtained from fitting CRTDs of UvrA-YPet in *uvrA-YPet* Δ*uvrB* cells. Shaded error bands are standard deviations from ten bootstrapped samples. Cartoon (inset) illustrates the arrested complex formed by UvrA-YPet (purple) and Mfd (green) in *uvrA-YPet* Δ*uvrB* cells.

c. Maximum intensity projection of *mfd-PAmCherry1* cells obtained upon exposure to 405-nm and 568-nm light.

d. Maximum intensity projection of *uvrA-PAmCherry1* cells obtained upon exposure to 405-nm and 568-nm light.

Note the significantly fewer localizations obtained in these strains compared to the YPet tagged constructs (Figure 2) and reference 1. Cell outlines (yellow) are provided as guide to the eye. Scale bar represents 5 μm. Source data are provided as a Source Data file.

Supplementary Figure 4:

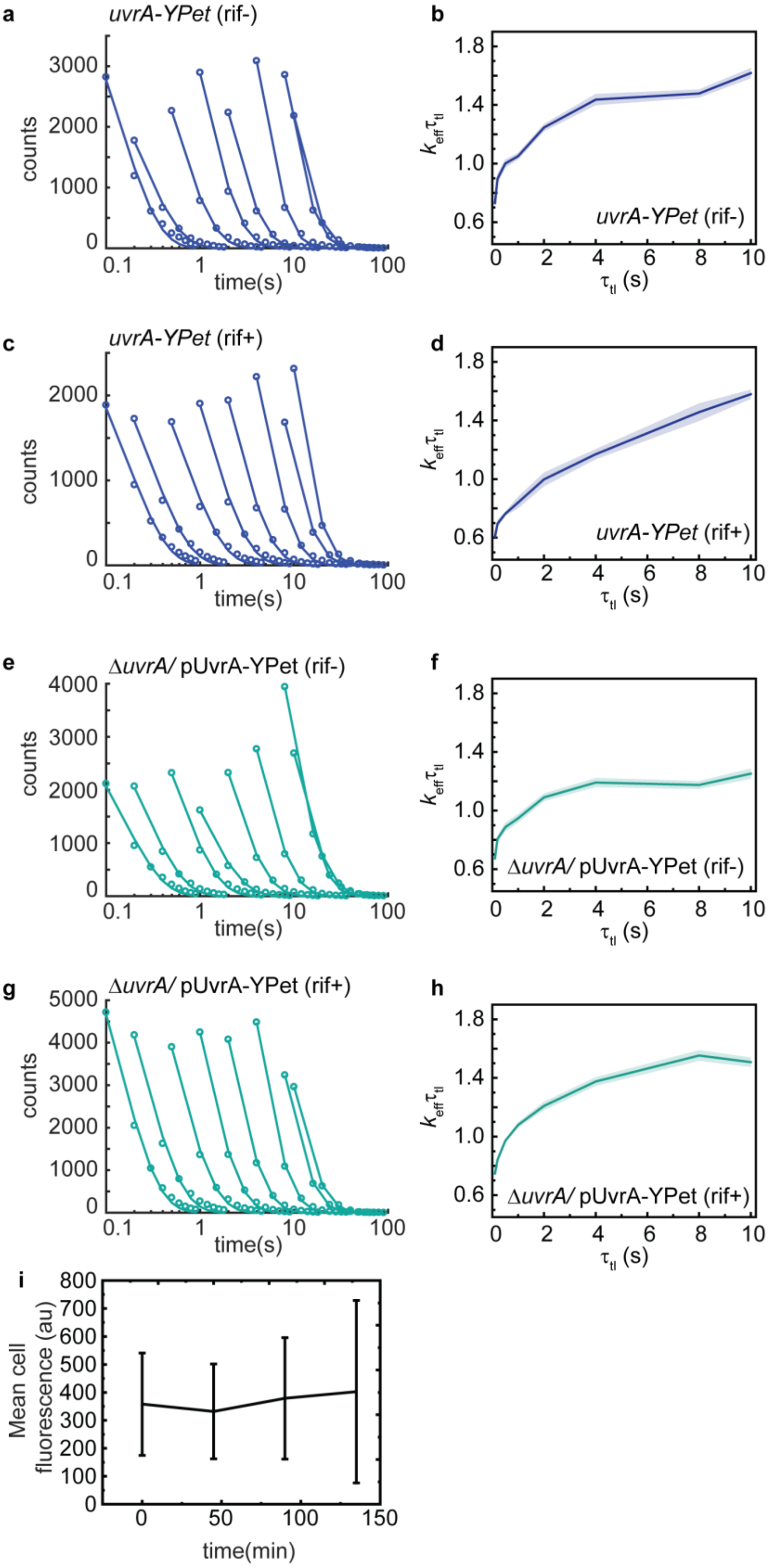

Supplementary Figure 4:
a. CRTDs (circles) obtained from interval imaging of UvrA-YPet in *uvrA-YPet* cells. Lines are mono-exponential fits to CRTDs.

b. The $k_{eff}\tau_{tl}$ plot obtained from fitting CRTDs of UvrA-YPet in *uvrA-YPet* cells. Shaded error bands are standard deviations from ten bootstrapped samples.

c. CRTDs (circles) obtained from interval imaging of UvrA-YPet in rif-treated *uvrA-YPet* cells. Lines are mono-exponential fits to CRTDs.

d. The $k_{eff}\tau_{tl}$ plot obtained from fitting CRTDs of UvrA-YPet in rif-treated *uvrA-YPet* cells. Shaded error bands are standard deviations from ten bootstrapped samples.

e. CRTDs (circles) obtained from interval imaging of UvrA-YPet in Δ*uvrA*/pUvrA-YPet cells. Lines are mono-exponential fits to CRTDs.

f. The $k_{eff}\tau_{tl}$ plot obtained from fitting CRTDs of UvrA-YPet in Δ*uvrA*/pUvrA-YPet cells. Shaded error bands are standard deviations from ten bootstrapped samples.

g. CRTDs (circles) obtained from interval imaging of UvrA-YPet in rif-treated Δ*uvrA*/pUvrA-YPet cells. Lines are mono-exponential fits to CRTDs.

h. The $k_{eff}\tau_{tl}$ plot obtained from fitting CRTDs of UvrA-YPet in rif-treated Δ*uvrA*/pUvrA-YPet cells. Shaded error bands are standard deviations from ten bootstrapped samples.

i. Mean pixel intensity (corrected for background) and standard deviation of all pixel intensities in cells (between 50 – 150 cells were analyzed at the indicated time points t = 0, 45, 90 and 135 min) following 30 min rif treatment. Cell fluorescence remains constant within error of measurement.

Source data are provided as a Source Data file.

Supplementary Figure 5:

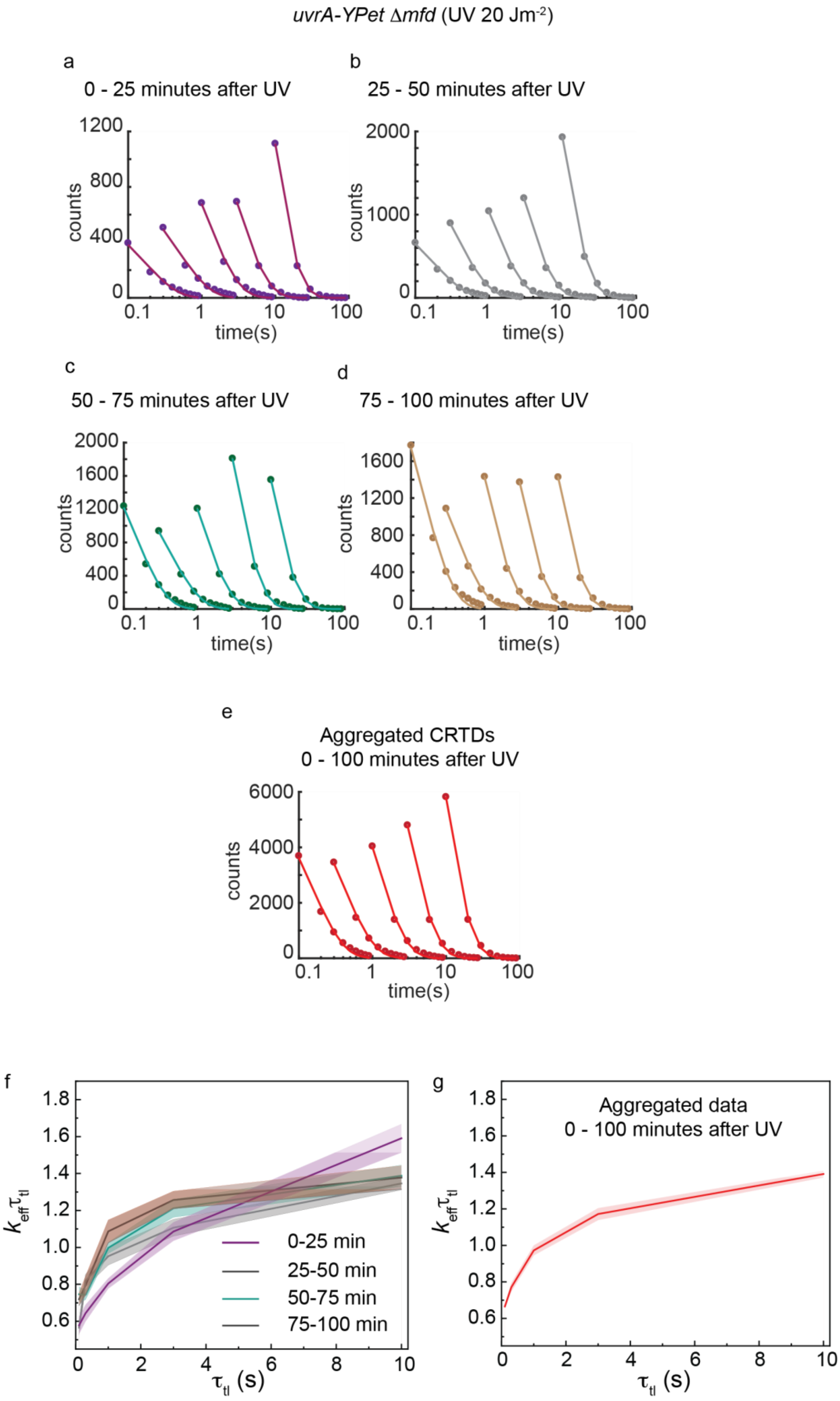

Supplementary Figure 5:
a. CRTDs (circles) obtained from interval imaging of UvrA-YPet in *uvrA-YPet* Δ*mfd* cells within the first 25 minutes following exposure to a 20 Jm^{-2} dose of 254-nm light. Lines are mono-exponential fits to CRTDs.

b. CRTDs (circles) obtained from interval imaging of UvrA-YPet in *uvrA-YPet* Δ*mfd* cells 25-50 minutes following exposure to a 20 Jm^{-2} dose of 254-nm light. Lines are mono-exponential fits to CRTDs.

c. CRTDs (circles) obtained from interval imaging of UvrA-YPet in *uvrA-YPet* Δ*mfd* cells 50-75 minutes following exposure to a 20 Jm^{-2} dose of 254-nm light. Lines are mono-exponential fits to CRTDs.

d. CRTDs (circles) obtained from interval imaging of UvrA-YPet in *uvrA-YPet* Δ*mfd* cells 75-100 minutes following exposure to a 20 Jm^{-2} dose of 254-nm light. Lines are mono-exponential fits to CRTDs.

e. CRTDs (circles) obtained from interval imaging of UvrA-YPet in *uvrA-YPet* Δ*mfd* cells within the first 100 minutes following exposure to a 20 Jm^{-2} dose of 254-nm light. Lines are mono-exponential fits to CRTDs.

f. The $k_{eff}\tau_{tl}$ plots obtained from fitting CRTDs of UvrA-YPet in UV-treated *uvrA-YPet* Δ*mfd* cells as a function of time following UV exposure. Shaded error bands are standard deviations from ten bootstrapped samples. Purple, 0-25 minutes; grey, 25-50 minutes; cyan, 50-75 minutes; brown, 75-100 minutes.

g. The $k_{eff}\tau_{tl}$ plot obtained from fitting CRTDs of UvrA-YPet in UV-treated *uvrA-YPet* Δ*mfd* cells within the first 100 minutes following UV exposure.
Source data are provided as a Source Data file.

Supplementary Figure 6:

uvrA-YPet **(UV 20 Jm^{-2})**

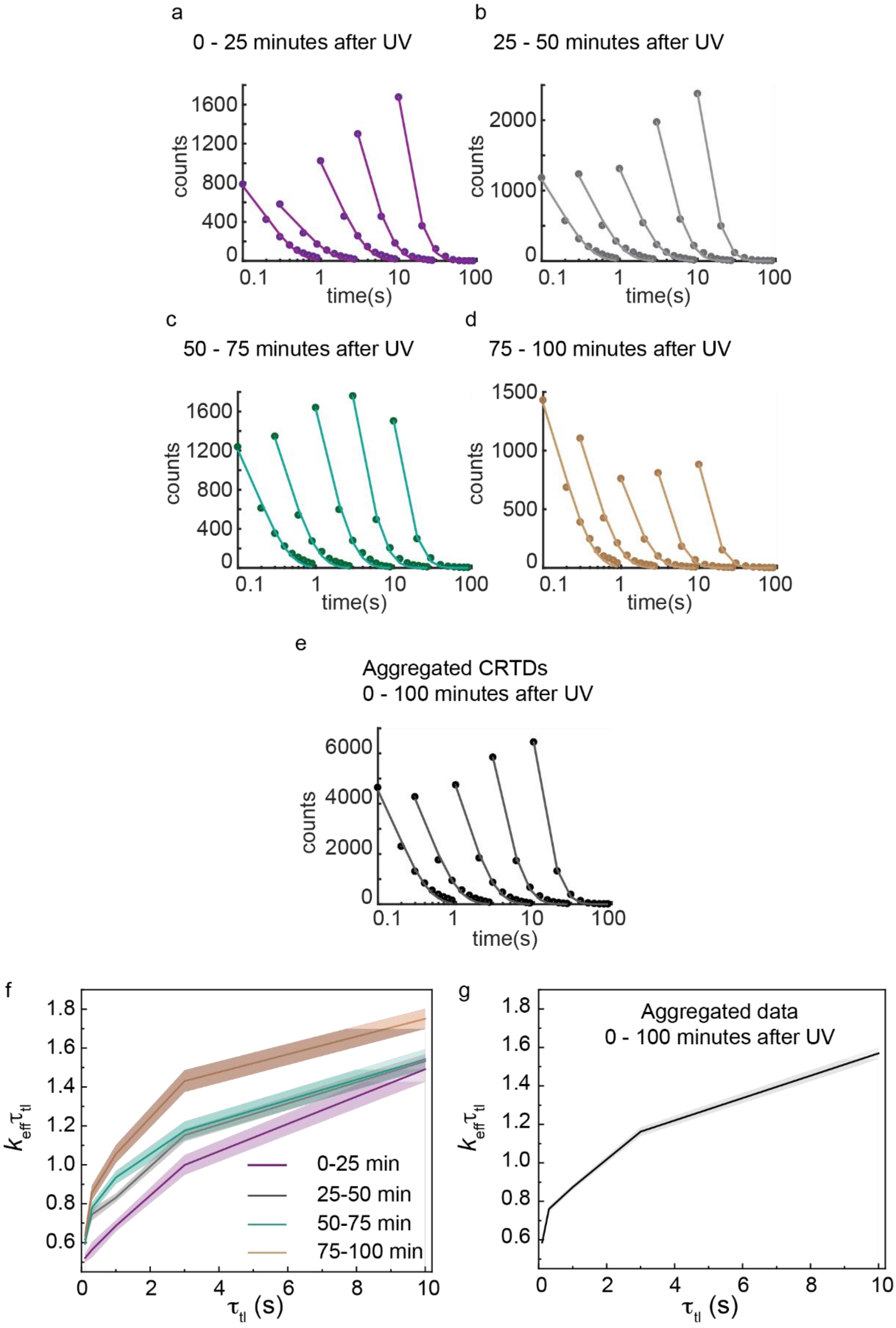

Supplementary Figure 6:
a. CRTDs (circles) obtained from interval imaging of UvrA-YPet in *uvrA-YPet* cells within the first 25 minutes following exposure to a 20 Jm^{-2} dose of 254-nm light. Lines are mono-exponential fits to CRTDs.

b. CRTDs (circles) obtained from interval imaging of UvrA-YPet in *uvrA-YPet* cells 25-50 minutes following exposure to a 20 Jm^{-2} dose of 254-nm light. Lines are mono-exponential fits to CRTDs.

c. CRTDs (circles) obtained from interval imaging of UvrA-YPet in *uvrA-YPet* cells 50-75 minutes following exposure to a 20 Jm^{-2} dose of 254-nm light. Lines are mono-exponential fits to CRTDs.

d. CRTDs (circles) obtained from interval imaging of UvrA-YPet in *uvrA-YPet* cells 75-100 minutes following exposure to a 20 Jm^{-2} dose of 254-nm light. Lines are mono-exponential fits to CRTDs.

e. CRTDs (circles) obtained from interval imaging of UvrA-YPet in *uvrA-YPet* cells within the first 100 minutes following exposure to a 20 Jm^{-2} dose of 254-nm light. Lines are mono-exponential fits to CRTDs.

f. The $k_{eff}\,\tau_{tl}$ plots obtained from fitting CRTDs of UvrA-YPet in UV-treated *uvrA-YPet* cells as a function of time following UV exposure. Shaded error bands are standard deviations from ten bootstrapped samples. Purple, 0-25 minutes; grey, 25-50 minutes; cyan, 50-75 minutes; brown, 75-100 minutes.

g. The $k_{eff}\,\tau_{tl}$ plot obtained from fitting CRTDs of UvrA-YPet in UV-treated *uvrA-YPet* cells within the first 100 minutes following UV exposure.
Source data are provided as a Source Data file.

Supplementary Figure 7:

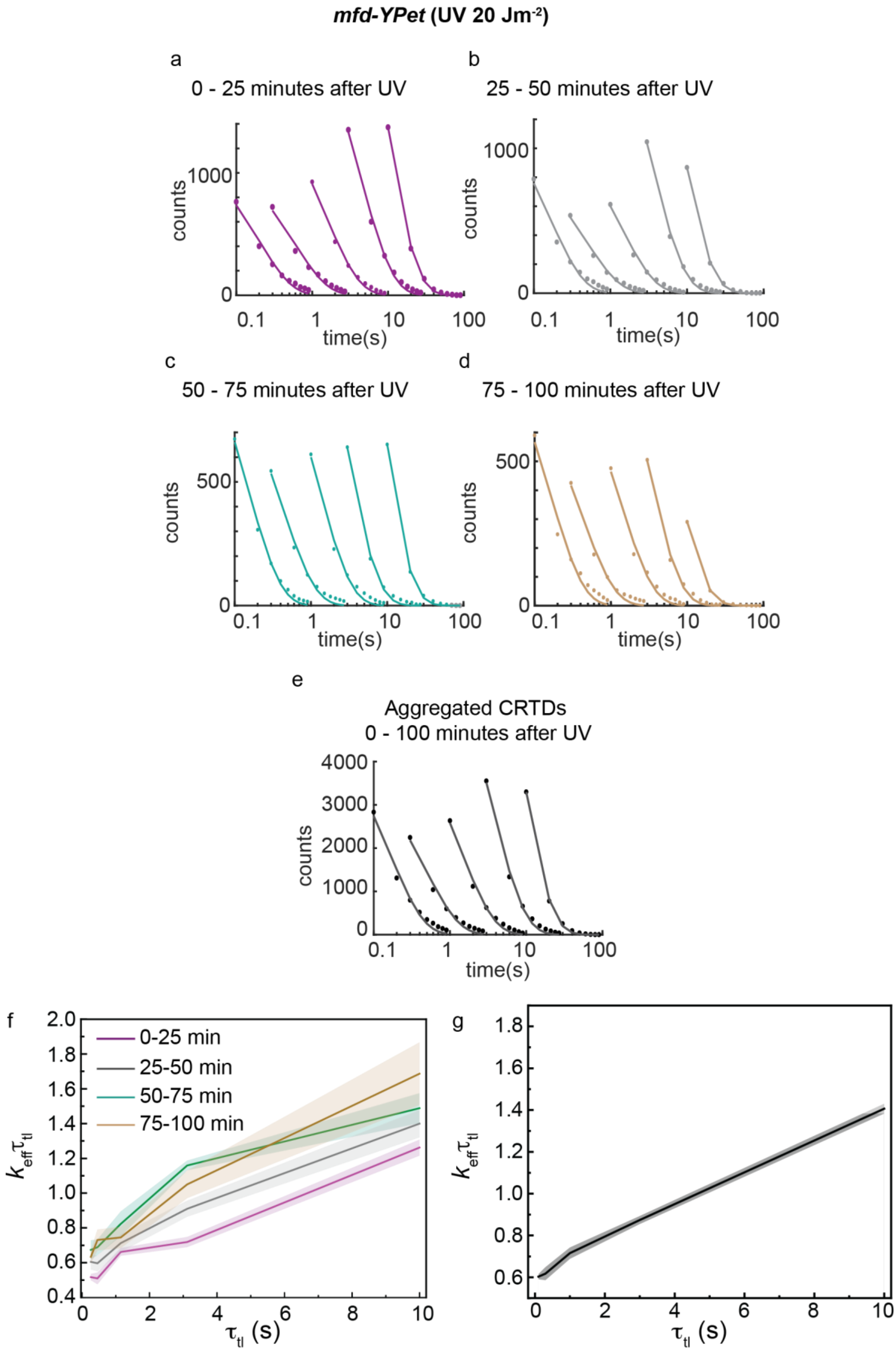

Supplementary Figure 7:
a. CRTDs (circles) obtained from interval imaging of Mfd-YPet in *mfd-YPet* cells within the first 25 minutes following exposure to a 20 Jm^{-2} dose of 254-nm light. Lines are mono-exponential fits to CRTDs.

b. CRTDs (circles) obtained from interval imaging of Mfd-YPet in *mfd-YPet* cells 25-50 minutes following exposure to a 20 Jm^{-2} dose of 254-nm light. Lines are mono-exponential fits to CRTDs.

c. CRTDs (circles) obtained from interval imaging of Mfd-YPet in *mfd-YPet* cells 50-75 minutes following exposure to a 20 Jm^{-2} dose of 254-nm light. Lines are mono-exponential fits to CRTDs.

d. CRTDs (circles) obtained from interval imaging of Mfd-YPet in *mfd-YPet* cells 75-100 minutes following exposure to a 20 Jm^{-2} dose of 254-nm light. Lines are mono-exponential fits to CRTDs.

e. CRTDs (circles) obtained from interval imaging of Mfd-YPet in *mfd-YPet* cells within the first 100 minutes following exposure to a 20 Jm^{-2} dose of 254-nm light. Lines are mono-exponential fits to CRTDs.

f. The $k_{\mathrm{eff}}\tau_{\mathrm{tl}}$ plots obtained from fitting CRTDs of Mfd-YPet in UV-treated *mfd-YPet* cells as a function of time following UV exposure. Shaded error bands are standard deviations from ten bootstrapped samples. Purple, 0-25 minutes; grey, 25-50 minutes; cyan, 50-75 minutes; brown, 75-100 minutes.

g. The $k_{\mathrm{eff}}\tau_{\mathrm{tl}}$ plot obtained from fitting CRTDs of Mfd-YPet in UV-treated *mfd-YPet* cells within the first 100 minutes following UV exposure.
Source data are provided as a Source Data file.

Supplementary Tables

Supplementary Table 1. Bacterial strains. All strains are in *E. coli* K-12 MG1655 background.

**Strain/genotypes**	**Source/Technique**
uvrA-YPet	This study/ λ Red recombination
uvrA-PAmCherry1	This study/ λ Red recombination
uvrA::kanR	This study
uvrB::kanR	This study/ λ Red recombination
mfd::kanR	This laboratory
ΔuvrA Δmfd ΔuvrB	This study
*ΔuvrA/*pUvrA-YPet	This study
*ΔuvrA Δmfd ΔuvrB/*pUvrA-YPet	This study
ΔuvrA Δmfd ΔuvrB/ pUvrA(Δ131-250)-YPet	This study
uvrA-YPet Δmfd	This study/ P1 transduction
uvrA-YPet uvrB(ΔβHG)	This study/ CRISPR-Cas9 assisted λ Red recombination
uvrA-YPet uvrB(ΔβHG) Δmfd	This study/ P1 transduction
uvrA-YPet ΔuvrB	This study/ P1 transduction
mfd-YPet	This laboratory
mfd-PAmCherry1	This study

Supplementary Table 2. Oligonucleotides used for colony PCR and CRISPR-Cas9 assisted λ Red recombination.

Oligo names	Sequence
UvrA_YPet_fw	TCGCG GAGTG CGAAG CATCA CACAC GGCAC GCTTC CTTAA GCCGA TGCTG TCGGCTGGCTCCGCTGCTGGTTCTGGCGAATTC ATG TCT AAA GGT GAA GAA TTA TTC ACT GGT G
UvrA_rev	GCTGG TGCAA CTCTG AAAGG AAAAG GCCGC TCAGA AAGCG GCCTT AACGA GAA GTT CCT ATT CTC TAG AAA GTA TAG G
UvrA_del_507_fw	CGGTA GCACC ATGCC ACCGG GCAAA AAAGC GTTTA ATCCG GGAAA GGTGA ccctttcgtcttcaagaattc
UvrA_del_507_rev	GCTGG TGCAA CTCTG AAAGG AAAAG GCCGC TCAGA AAGCG GCCTT AACGA ggccacgatgcgtccggcgta
uvrA_del_seq_fw1	AACCTGGCCAGACATTGTTAC
uvrA_del_seq_rev1	CTGTTTGCGATGGTGAAAGA
UvrB_del_seq_fw	CCC AAC TCC TTC AGG TAG CG
UvrB_del_seq_rev	TAG GCC TGA TAA GCG TAG CG
UvrA_FWc	TGGGTACCGGGCCCGCGATTGTACCATTACCAATAG
UvrAYPet_revc	AGCCAGCCGAagcgatcgcCAGCATCGGCTTAAGGA
RecASZ2	GTGGCGGCCGCTCTA
Colony PCR for Cas9 verification	
dCas9dL5_303_F	CAGACCGCCACAGTATCAAA
pCas9_6700_R	GGAAGGTATCCGACTGCTG
Cloning of pCRISPR variants	
pCRISPR_UvrB_Y96A_S	AAA CCC TAC TAC GAC TAC TAT CAG CG
pCRISPR_UvrB_Y96A_AS	AAA ACG CTG ATA GTA GTC GTA GTA GG
Recombineric ssDNA	
UvrB_ΔβHG_ ssDNA	CAA TAT GTT CGT TAA CCG AGG CAT CTT TCT CAA TGA AAG TGC CAT AAT AGT CGT AGT AGG AAA CGA AAT ATT CCA CCG CGT TTT CCG

Supplementary Table 3. Plasmids.

Plasmid	Source/Technique
pHH001 (pSC101-based)	Plasmid backbone carrying a pSC101 origin, spectinomycin marker and expresses Mfd-YPet under the native *mfd* promoter[1]. pJM1071 used as a backbone was a generous gift from the Woodgate laboratory.[2]
pHH002	Plasmid backbone carrying a pSC101 origin, spectinomycin marker and expresses Mfd-YPet under the native *mfd* promoter. This lab[1].
pYPet	This lab (synthetic gene obtained from GeneArt AG)
pUvrA-YPet	This study/Sub-cloning into pHH001
pUvrA(Δ131-250)-YPet	This study/Sub-cloning into pHH002
pKD46	Cox lab[3]
pCas9	Addgene # 42876, Marraffini lab[4]
pCRISPR	Addgene # 42875, Marraffini lab[4]
pCRISPR-UvrB-Y96A	This study/Sub-cloning into pCRISPR

Supplementary Table 4: Sequences of plasmids and geneblocks used in this work

Plasmid	Sequence
pEAW507	cgaggccctttcgtcttcaagaattcGAAGTTCCTATAGTTTCTAGAGAATAGGAACTTCgatcttt<br>agaaaaactcatcgagcatcaaatgaaactgcaatttattcatatcaggattatcaataccatatttttgaaaaag<br>ccgtttctgtaatgaaggagaaaactcaccgaggcagttccataggatggcaagatcctggtatcggtctgcgatt<br>ccgactcgtccaacatcaatacaacctattaatttcccctcgtcaaaaataaggttatcaagtgagaaatcaccat<br>gagtgacgactgaatccggtgagaatggcaaaagcttatgcatttctttccagacttgttcaacaggccagccatt<br>acgctcgtcatcaaaatcactcgcatcaaccaaaccgttattcattcgtgattgcgcctgagcgagacgaaatacg<br>cgatcgctgttaaaaggacaattacaaacaggaatcgaatgcaaccggcgcaggaacactgccagcgcatcaac<br>aatattttcacctgaatcaggatattcttctaatacctggaatgctgttttcccggggatcgcagtggtgagtaacca<br>tgcatcatcaggagtacggataaaatgcttgatggtcggaagaggcataaattccgtcagccagtttagtctgacc<br>atctcatctgtaacatcattggcaacgctacctttgccatgtttcagaaacaactctggcgcatcgggcttcccatac<br>aatcgatagattgtcgcacctgattgcccgacattatcgcgagcccatttatacccatataaatcagcatccatgtt<br>ggaatttaatcgcggcctcgagcaagacgtttcccgttgaatatggctcataacaccccttgtattactgtttatgta<br>agcagacagttttattgttcatgatgatatatttttatcttgtgcaatgtaacatcagagattttgagacacaacgtg<br>gctttccccccccccccgatccccgggtaccgagctcgaatttcgaccaattcGAAGTTCCTATACTTTCTA<br>GAGAATAGGAACTTCcgggatcctctacgccggacgcatcgtggccggcatcaccggcgccacaggtgcg<br>gttgctggcgcctatatcgccgacatcaccgatggggaagatcgggctcgccacttcgggctcatgagcgcttgt<br>ttcggcgtgggtatggtggcaggccccgtggccgggggactgttgggcgccatctccttgcatgcaccattccttgc<br>ggcggcggtgctcaacggcctcaacctactactggcgctgcttcctaatgcaggagtcgcataagggagagcgtc<br>gaccgatgcccttgagagccttcaacccagtcagctccttccggtgggcgcggggcatgactatcgtcgccgcact<br>tatgactgtcttctttatcatgcaactcgtaggacaggtgccggcagcgctctgggtcattttcggcgaggaccgctt<br>tcgctggagcgcgacgatgatcggcctgtcgcttgcggtattcggaatcttgcacgccctcgctcaagccttcgtca<br>ctggtcccgccaccaaacgtttcggcgagaagcaggccattatcgccggcatggcggccgacgcgctgggctacg<br>tcttgctggcgttcgcgacgcgaggctggatggccttccccattatgattcttctcgcttccggcggcatcgggatgc<br>ccgcgttgcaggccatgctgtccaggcaggtagatgacgaccatcagggacagcttcaaggatcgctcgcggctc<br>ttaccagcctaacttcgatcactggaccgctgatcgtcacggcgatttatgccgcctcggcgagcacatggaacgg<br>gttggcatggattgtaggcgccgccctataccttgtctgcctccccgcgttgcgtcgcggtgcatggagccgggcca<br>cctcgacctgaatggaagccggcggcacctcgctaacggattcaccactccaagaattggagccaatcaattcttg<br>cggagaactgtgaatgcgcaaaccaacccttggcagaacatatccatcgcgtccgccatctccagcagccgcacg

	cggcgcatcgcgttgctggcgtttttccataggctccgcccccctgacgagcatcacaaaaatcgacgctcaagtc<br>agaggtggcgaaacccgacaggactataaagataccaggcgtttccccctggaagctccctcgtgcgctctcctgt<br>tccgaccctgccgcttaccggatacctgtccgcctttctcccttcgggaagcgtggcgctttctcatagctcacgctg<br>taggtatctcagttcggtgtaggtcgttcgctccaagctgggctgtgtgcacgaaccccccgttcagcccgaccgct<br>gcgccttatccggtaactatcgtcttgagtccaacccggtaagacacgacttatcgccactggcagcagccactgg<br>taacaggattagcagagcgaggtatgtaggcggtgctacagagttcttgaagtggtggcctaactacggctacac<br>tagaaggacagtatttggtatctgcgctctgctgaagccagttaccttcggaaaaagagttggtagctcttgatccg<br>gcaaacaaaccaccgctggtagcggtggtttttttgtttgcaagcagcagattacgcgcagaaaaaaaggatctc<br>aagaagatcctttgatcttttctacggggtctgacgctcagtggaacgaaaactcacgttaagggattttggtcatg<br>agattatcaaaaaggatcttcacctagatccttttaaattaaaaatgaagttttaaatcaatctaaagtatatatga<br>gtaaacttggtctgacagttaccaatgcttaatcagtgaggcacctatctcagcgatctgtctatttcgttcatccat<br>agttgcctgactccccgtcgtgtagataactacgatacgggagggcttaccatctggccccagtgctgcaatgata<br>ccgcgagacccacgctcaccggctccagatttatcagcaataaaccagccagccggaagggccgagcgcagaa<br>gtggtcctgcaactttatccgcctccatccagtctattaattgttgccgggaagctagagtaagtagttcgccagtta<br>atagtttgcgcaacgttgttgccattgctgcaggcatcgtggtgtcacgctcgtcgtttggtatggcttcattcagctc<br>cggttcccaacgatcaaggcgagttacatgatcccccatgttgtgcaaaaaagcggttagctccttcggtcctccg<br>atcgttgtcagaagtaagttggccgcagtgttatcactcatggttatggcagcactgcataattctcttactgtcatg<br>ccatccgtaagatgcttttctgtgactggtgagtactcaaccaagtcattctgagaatagtgtatgcggcgaccgag<br>ttgctcttgcccggcgtcaacacgggataataccgcgccacatagcagaactttaaaagtgctcatcattggaaaa<br>cgttcttcggggcgaaaactctcaaggatcttaccgctgttgagatccagttcgatgtaacccactcgtgcacccaa<br>ctgatcttcagcatcttttactttcaccagcgtttctgggtgagcaaaaacaggaaggcaaaatgccgcaaaaaag<br>ggaataagggcgacacggaaatgttgaatactcatactcttcctttttcaatattattgaagcatttatcagggttat<br>tgtctcatgagcggatacatatttgaatgtatttagaaaaataaacaaataggggttccgcgcacatttccccgaa<br>aagtgccacctgacgtctaagaaaccattattatcatgacattaacctataaaaataggcgtatca
pYPet	CTAAATTGTAAGCGTTAATATTTTGTTAAAATTCGCGTTAAATTTTTGTTAAATCAGCTC<br>ATTTTTTAACCAATAGGCCGAAATCGGCAAAATCCCTTATAAATCAAAAGAATAGACCG<br>AGATAGGGTTGAGTGGCCGCTACAGGGCGCTCCCATTCGCCATTCAGGCTGCGCAACT<br>GTTGGGAAGGGCGTTTCGGTGCGGGCCTCTTCGCTATTACGCCAGCTGGCGAAAGGG<br>GGATGTGCTGCAAGGCGATTAAGTTGGGTAACGCCAGGGTTTTCCCAGTCACGACGTT<br>GTAAAACGACGGCCAGTGAGCGCGACGTAATACGACTCACTATAGGGCGAATTGGCG<br>GAAGGCCGTCAAGGCCGCATATGTCTAAAGGTGAAGAATTATTCACTGGTGTTGTCCC

	AATTTTGGTTGAATTAGATGGTGATGTTAATGGTCACAAATTTTCTGTCTCCGGTGAAG GTGAAGGTGATGCTACGTACGGTAAATTGACCTTAAAATTACTCTGTACTACTGGTAA ATTGCCAGTTCCATGGCCAACCTTAGTCACTACTTTAGGTTATGGTGTTCAATGTTTTGC TAGATACCCAGATCATATGAAACAACATGACTTTTTCAAGTCTGCCATGCCAGAAGGTT ATGTTCAAGAAAGAACTATTTTTTTCAAAGATGACGGTAACTACAAGACCAGAGCTGA AGTCAAGTTTGAAGGTGATACCTTAGTTAATAGAATCGAATTAAAAGGTATTGATTTTA AAGAAGATGGTAACATTTTAGGTCACAAATTGGAATACAACTATAACTCTCACAATGTT TACATCACTGCTGACAAACAAAAGAATGGTATCAAAGCTAACTTCAAAATTAGACACA ACATTGAAGATGGTGGTGTTCAATTAGCTGACCATTATCAACAAAATACTCCAATTGGT GATGGTCCAGTCTTGTTACCAGACAACCATTACTTATCCTATCAATCTGCCTTATTCAAA GATCCAAACGAAAAGAGAGACCACATGGTCTTGTTAGAATTTTTGACTGCTGCTGGTA TTACCGAGGGTATGAATGAATTGTACAAAGAGCTCTAAGAAGTTCCTATAGTTTCTAG AGAATAGGAACTTCGATCTTTAGAAAAACTCATCGAGCATCAAATGAAACTGCAATTT ATTCATATCAGGATTATCAATACCATATTTTTGAAAAAGCCGTTTCTGTAATGAAGGAG AAAACTCACCGAGGCAGTTCCATAGGATGGCAAGATCCTGGTATCGGTCTGCGATTCC GACTCGTCCAACATCAATACAACCTATTAATTTCCCCTCGTCAAAAATAAGGTTATCAA GTGAGAAATCACCATGAGTGACGACTGAATCCGGTGAGAATGGCAAAAGCTTATGCA TTTCTTTCCAGACTTGTTCAACAGGCCAGCCATTACGCTCGTCATCAAAATCACTCGCAT CAACCAAACCGTTATTCATTCGTGATTGCGCCTGAGCGAGACGAAATACGCGATCGCT GTTAAAAGGACAATTACAAACAGGAATCGAATGCAACCGGCGCAGGAACACTGCCAG CGCATCAACAATATTTTCACCTGAATCAGGATATTCTTCTAATACCTGGAATGCTGTTTT CCCGGGGATCGCAGTGGTGAGTAACCATGCATCATCAGGAGTACGGATAAAATGCTT GATGGTCGGAAGAGGCATAAATTCCGTCAGCCAGTTTAGTCTGACCATCTCATCTGTA ACATCATTGGCAACGCTACCTTTGCCATGTTTCAGAAACAACTCTGGCGCATCGGGCTT CCCATACAATCGATAGATTGTCGCACCTGATTGCCCGACATTATCGCGAGCCCATTTAT ACCCATATAAATCAGCATCCATGTTGGAATTTAATCGCGGCCTCGAGCAAGACGTTTCC CGTTGAATATGGCTCATAACACCCCTTGTATTACTGTTTATGTAAGCAGACAGTTTTATT GTTCATGATGATATATTTTTATCTTGTGCAATGTAACATCAGAGATTTTGAGACACAAC GTGGCTTTCCCCCCCCCCCCGATCCCCGGGTACCGAGCTCGAATTTCGACCAATTCGAA GTTCCTATACTTTCTAGAGAATAGGAACTTCCTGGGCCTCATGGGCCTTCCGCTCACTG CCCGCTTTCCAGTCGGGAAACCTGTCGTGCCAGCTGCATTAACATGGTCATAGCTGTTT

	CCTTGCGTATTGGGCGCTCTCCGCTTCCTCGCTCACTGACTCGCTGCGCTCGGTCGTTC<br>GGGTAAAGCCTGGGGTGCCTAATGAGCAAAAGGCCAGCAAAAGGCCAGGAACCGTA<br>AAAAGGCCGCGTTGCTGGCGTTTTTCCATAGGCTCCGCCCCCCTGACGAGCATCACAA<br>AAATCGACGCTCAAGTCAGAGGTGGCGAAACCCGACAGGACTATAAAGATACCAGGC<br>GTTTCCCCCTGGAAGCTCCCTCGTGCGCTCTCCTGTTCCGACCCTGCCGCTTACCGGAT<br>ACCTGTCCGCCTTTCTCCCTTCGGGAAGCGTGGCGCTTTCTCATAGCTCACGCTGTAGG<br>TATCTCAGTTCGGTGTAGGTCGTTCGCTCCAAGCTGGGCTGTGTGCACGAACCCCCCGT<br>TCAGCCCGACCGCTGCGCCTTATCCGGTAACTATCGTCTTGAGTCCAACCCGGTAAGAC<br>ACGACTTATCGCCACTGGCAGCAGCCACTGGTAACAGGATTAGCAGAGCGAGGTATG<br>TAGGCGGTGCTACAGAGTTCTTGAAGTGGTGGCCTAACTACGGCTACACTAGAAGAAC<br>AGTATTTGGTATCTGCGCTCTGCTGAAGCCAGTTACCTTCGGAAAAAGAGTTGGTAGC<br>TCTTGATCCGGCAAACAAACCACCGCTGGTAGCGGTGGTTTTTTTGTTTGCAAGCAGCA<br>GATTACGCGCAGAAAAAAAGGATCTCAAGAAGATCCTTTGATCTTTTCTACGGGGTCT<br>GACGCTCAGTGGAACGAAAACTCACGTTAAGGGATTTTGGTCATGAGATTATCAAAAA<br>GGATCTTCACCTAGATCCTTTTAAATTAAAAATGAAGTTTTAAATCAATCTAAAGTATAT<br>ATGAGTAAACTTGGTCTGACAGTTACCAATGCTTAATCAGTGAGGCACCTATCTCAGC<br>GATCTGTCTATTTCGTTCATCCATAGTTGCCTGACTCCCCGTCGTGTAGATAACTACGAT<br>ACGGGAGGGCTTACCATCTGGCCCCAGTGCTGCAATGATACCGCGAGAACCACGCTCA<br>CCGGCTCCAGATTTATCAGCAATAAACCAGCCAGCCGGAAGGGCCGAGCGCAGAAGT<br>GGTCCTGCAACTTTATCCGCCTCCATCCAGTCTATTAATTGTTGCCGGGAAGCTAGAGT<br>AAGTAGTTCGCCAGTTAATAGTTTGCGCAACGTTGTTGCCATTGCTACAGGCATCGTG<br>GTGTCACGCTCGTCGTTTGGTATGGCTTCATTCAGCTCCGGTTCCCAACGATCAAGGCG<br>AGTTACATGATCCCCCATGTTGTGCAAAAAAGCGGTTAGCTCCTTCGGTCCTCCGATCG<br>TTGTCAGAAGTAAGTTGGCCGCAGTGTTATCACTCATGGTTATGGCAGCACTGCATAA<br>TTCTCTTACTGTCATGCCATCCGTAAGATGCTTTTCTGTGACTGGTGAGTACTCAACCAA<br>GTCATTCTGAGAATAGTGTATGCGGCGACCGAGTTGCTCTTGCCCGGCGTCAATACGG<br>GATAATACCGCGCCACATAGCAGAACTTTAAAAGTGCTCATCATTGGAAAACGTTCTTC<br>GGGGCGAAAACTCTCAAGGATCTTACCGCTGTTGAGATCCAGTTCGATGTAACCCACT<br>CGTGCACCCAACTGATCTTCAGCATCTTTTACTTTCACCAGCGTTTCTGGGTGAGCAAA<br>AACAGGAAGGCAAAATGCCGCAAAAAAGGGAATAAGGGCGACACGGAAATGTTGAA<br>TACTCATACTCTTCCTTTTTCAATATTATTGAAGCATTTATCAGGGTTATTGTCTCATGAG

	CGGATACATATTTGAATGTATTTAGAAAAATAAACAAATAGGGGTTCCGCGCACATTTC<br>CCCGAAAAGTGCCAC
UvrA d131_250 (gene block)	TGGGTACCGGGCCCGCGATTGTACCATTACCAATAGCGCTTTTACTATGTTGTGACCTC<br>GGTTCCGGGAAACAAACCTGGCCAGACATTGTTACACAACACTCCGGGTAATGCATTC<br>CAATACTGTATATTCATTCAGGTCAATTTGTGTCATAATTAACCGTTTGTGATCGCCGGT<br>AGCACCATGCCACCGGGCAAAAAAGCGTTTAATCCGGGAAAGGTGAATGGATAAGAT<br>CGAAGTTCGGGGCGCCCGCACCCATAATCTCAAAAACATCAACCTCGTTATCCCCCGCG<br>ACAAGCTCATTGTCGTGACCGGGCTTTCGGGTTCTGGCAAATCCTCGCTCGCTTTCGAC<br>ACCTTATATGCCGAAGGGCAGCGCCGTTACGTTGAATCCCTTTCCGCCTACGCGCGGC<br>AGTTTCTGTCACTGATGGAAAAGCCGGACGTCGATCATATTGAGGGGCTTTCTCCTGC<br>CATCTCAATTGAGCAGAAATCGACGTCTCATAACCCGCGTTCTACGGTGGGGACAATC<br>ACCGAAATCCACGACTATTTGCGTTTGTTATTCGCCCGCGTTGGCGAGCCGCGCTGTCC<br>GGACCACGACGTCCCGCTGGCGGCGCAAGGCTTCGCCTGCCCAATTTGCGGCTACAGT<br>ATGCGTGAACTGGAGCCGCGACTGTTTTCGTTTAACAACCCGGCGGGGGCCTGCCCGA<br>CCTGCGACGGCCTTGGCGTACAGCAATATTTCGATCCTGATCGAGTGATCCAGAATCC<br>GGAACTGTCGCTGGCTGGTGGTGCGATCCGTGGCTGGGATCGCCGCAACTTCTATTAT<br>TTCCAGATGCTGAAATCGCTGGCAGATCACTATAAGTTCGACGTCGAAGCGCCGTGGG<br>GCAGCCTGAGCGCGAACGTGCATAAAGTGGTGTTGTACGGTTCTGGCAAAGAAAACA<br>TTGAATTCAAATACATGAACGATCGTGGCGATACCTCCATTCGTCGTCATCCGTTCGAA<br>GGCGTGCTGCATAATATGGAGCGCCGCTATAAAGAGACGGAATCCAGCGCGGTACGC<br>GAAGAATTAGCCAAGTTTATCAGTAATCGTCCGTGCGCCAGCTGCGAAGGGACGCGTC<br>TGCGTCGGGAAGCGCGCCACGTGTATGTCGAGAATACGCCGCTGCCTGCTATCTCCGA<br>CATGAGCATTGGTCATGCGATGGAATTCTTCAACAATCTCAAACTCGCAGGTCAGCGG<br>GCGAAGATTGCAGAAAAAATCCTTAAAGAGATCGGCGATCGTCTGAAATTCCTCGTTA<br>ACGTCGGCCTGAATTACCTGACGCTTTCCCGCTCGGCAGAAACGCTTTCTGGCGGTGA<br>AGCACAGCGTATCCGTCTGGCGAGCCAGATTGGTGCGGGCCTGGTTGGCGTTATGTAC<br>GTGCTGGACGAGCCGTCTATCGGCCTGCACCAGCGTGATAACGAGCGCCTGTTGGGT<br>ACGCTTATCCATCTGCGCGATCTCGGTAATACCGTGATTGTGGTGGAGCACGACGAAG<br>ACGCAATTCGCGCCGCTGACCATGTGATCGACATTGGCCCGGGCGCAGGTGTTCACGG<br>CGGTGAAGTGGTCGCAGAAGGTCCGCTGGAAGCGATTATGGCGGTGCCGGAGTCGTT<br>GACCGGGCAGTACATGAGCGGCAAACGCAAGATTGAAGTGCCGAAGAAACGCGTTCC

	GGCGAATCCGGAAAAAGTGCTGAAGCTGACAGGCGCACGCGGCAACAACCTGAAGG<br>ACGTGACGCTGACGCTGCCGGTGGGTCTGTTTACCTGCATCACCGGGGTTTCAGGTTC<br>CGGTAAATCGACGCTGATTAACGACACACTGTTCCCGATTGCCCAACGCCAGTTGAAT<br>GGGGCGACCATCGCCGAACCAGCACCGTATCGCGATATTCAGGGGCTGGAGCATTTC<br>GATAAAGTGATCGATATCGACCAAAGCCCAATTGGTCGTACTCCACGTTCTAACCCGG<br>CGACCTATACCGGCGTGTTTACGCCTGTGCGCGAACTGTTTGCGGGCGTACCGGAATC<br>CCGTGCGCGCGGCTATACGCCGGGACGTTTCAGCTTTAACGTTCGTGGCGGACGCTGC<br>GAGGCCTGTCAGGGCGATGGCGTGATCAAAGTGGAGATGCACTTCCTGCCGGATATC<br>TACGTGCCGTGCGACCAGTGCAAAGGTAAACGCTATAACCGTGAAACGCTGGAGATT<br>AAGTACAAAGGCAAAACCATCCACGAAGTGCTGGATATGACCATCGAAGAGGCGCGT<br>GAGTTCTTTGATGCCGTACCTGCACTGGCGCGTAAGCTGCAAACGTTGATGGACGTTG<br>GCCTGACGTACATTCGACTGGGGCAGTCCGCAACCACCCTTTCAGGCGGTGAAGCCCA<br>GCGCGTGAAGCTGGCGCGTGAACTGTCAAAACGCGGCACCGGGCAGACGCTGTATAT<br>TCTCGACGAGCCGACCACCGGTCTGCACTTCGCCGATATTCAGCAACTGCTCGACGTAC<br>TGCATAAACTGCGCGATCAGGGCAACACCATTGTGGTGATTGAGCACAATCTCGACGT<br>GATCAAAACCGCTGACTGGATTGTCGACCTGGGACCAGAAGGCGGCAGTGGTGGCGG<br>CGAGATCCTCGTCTCCGGTACGCCAGAAACCGTCGCGGAGTGCGAAGCATCACACACG<br>GCACGCTTCCTTAAGCCGATGCTGGCGATCGCTTCGGCTGGCTCCGCTGCAGGTTCTG<br>GCGAATTCGGGTAATGATTCAGTTCTAGAGCGGCCGCCACTAA

Supplementary Notes

Supplementary Note 1

The chromosomal fusions of *uvrA-YPet* and *uvrA-PAmCherry1* and *mfd-PAmCherry1* were created using λ Red recombination as previously described and the primers indicated in supplementary table 2[3]. Sequence verified recombinant strains carrying Mfd-PAmCherry1 and UvrA-PAmCherry1 were then screened by imaging at the single-cell level. Unlike their YPet tagged counterparts, the PAmCherry1 labelled proteins were poorly expressed in MG1655 (Supplementary Fig. 3).

Supplementary Note 2: Copy numbers of UvrA after SOS induction

UvrA copy numbers have been suggested to increase from 25 to 250 copies per cell after SOS induction[5]. We note that since the experimental conditions associated with these measurements are not available in the published literature, we are unable to effectively compare our measurements with these numbers. Nevertheless, the basal levels and the exact extent of fold-induction after SOS induction may depend on the nature and dosage of the genotoxin, as well as growth conditions such as medium and temperature. Consistent with this argument, the copy numbers of UvrA were found to rise six-fold within 40 minutes of UV exposure (40 Jm^{-2}) in cells growing at 37 °C in previous work [6].

Supplementary References

1 Ho HN, van Oijen AM, Ghodke H. The transcription-repair coupling factor Mfd associates with RNA polymerase in the absence of exogenous damage. *Nat Commun* **9**, 1570 (2018).

2 Churchward G, Belin D, Nagamine Y. A pSC101-derived plasmid which shows no sequence homology to other commonly used cloning vectors. *Gene* **31**, 165-171 (1984).

3 Datsenko KA, Wanner BL. One-step inactivation of chromosomal genes in *Escherichia coli* K-12 using PCR products. *Proc Natl Acad Sci U S A* **97**, 6640-6645 (2000).

4 Jiang W, Bikard D, Cox D, Zhang F, Marraffini LA. RNA-guided editing of bacterial genomes using CRISPR-Cas systems. *Nat Biotechnol* **31**, 233-239 (2013).

5 Selby, C. P. & Sancar, A. Structure and function of the (A)BC excinuclease of *Escherichia coli*. *Mutat Res* **236**, 203-211 (1990).

6 Crowley DJ, Hanawalt PC. Induction of the SOS Response Increases the Efficiency of Global Nucleotide Excision Repair of Cyclobutane Pyrimidine Dimers, but Not 6-4 Photoproducts, in UV-Irradiated *Escherichia coli*. *J Bacteriol* **180**, 3345-3352 (1998).
