# Supplementary material for: Single-molecule live-cell imaging visualizes parallel pathways of prokaryotic nucleotide excision repair
Source: Nat Commun. 2020 Mar 20;11:1477. doi: 10.1038/s41467-020-15179-y (PMC7083872; doi:10.1038/s41467-020-15179-y)
Supplement: Supplementary file 2 — Description of Additional Supplementary Files [file 41467_2020_15179_MOESM2_ESM.pdf]

Description of Additional Supplementary Files

File Name: Supplementary Movie 1

Description: **Imaging of UvrA-YPet cells.** Movie showing UvrA-YPet cells immobilized in a flow cell and exposed to 514 nm laser light. Acquisition is collected in two phases. Phase I consists of 50 frames of 100 ms each. Phase II consists of 100 frames of 100 ms each. Frames in phase II are collected by introduction of a dark interval. Here, example video shows continuous acquisition in both phases. Scale bar represents 5 μm.
